# ConanVarvar: a versatile tool for the detection of large syndromic copy number variation from whole-genome sequencing data

**DOI:** 10.1186/s12859-023-05154-x

**Published:** 2023-02-15

**Authors:** Mikhail Gudkov, Loïc Thibaut, Matloob Khushi, Gillian M. Blue, David S. Winlaw, Sally L. Dunwoodie, Eleni Giannoulatou

**Affiliations:** 1 Victor Chang Cardiac Research Institute, Sydney, NSW 2010 Australia; 2 School of Biomedical Engineering, The University of Sydney, Sydney, NSW 2006 Australia; 3 St Vincent’s Clinical Campus, School of Clinical Medicine, Faculty of Medicine and Health, UNSW Sydney, Sydney, NSW 2010 Australia; 4 School of Mathematics and Statistics, UNSW Sydney, Sydney, NSW 2052 Australia; 5 School of Computer Science, The University of Sydney, Sydney, NSW 2006 Australia; 6 Sydney Medical School, The University of Sydney, Sydney, NSW 2006 Australia; 7 Heart Centre for Children, The Children’s Hospital at Westmead, Sydney, NSW 2145 Australia; 8 School of Biotechnology and Biomolecular Sciences, UNSW Sydney, Sydney, NSW 2052 Australia

**Keywords:** Bioinformatics, WGS, CNV, Docker

## Abstract

**Background:**

A wide range of tools are available for the detection of copy number variants (CNVs) from whole-genome sequencing (WGS) data. However, none of them focus on clinically-relevant CNVs, such as those that are associated with known genetic syndromes. Such variants are often large in size, typically 1–5 Mb, but currently available CNV callers have been developed and benchmarked for the discovery of smaller variants. Thus, the ability of these programs to detect tens of real syndromic CNVs remains largely unknown.

**Results:**

Here we present ConanVarvar, a tool which implements a complete workflow for the targeted analysis of large germline CNVs from WGS data. ConanVarvar comes with an intuitive R Shiny graphical user interface and annotates identified variants with information about 56 associated syndromic conditions. We benchmarked ConanVarvar and four other programs on a dataset containing real and simulated syndromic CNVs larger than 1 Mb. In comparison to other tools, ConanVarvar reports 10–30 times less false-positive variants without compromising sensitivity and is quicker to run, especially on large batches of samples.

**Conclusions:**

ConanVarvar is a useful instrument for primary analysis in disease sequencing studies, where large CNVs could be the cause of disease.

**Supplementary Information:**

The online version contains supplementary material available at 10.1186/s12859-023-05154-x.

## Background

A copy number variant (CNV) is defined as a DNA fragment with a size of at least 1 kilobase-pair (kb) which has a different copy number compared to a reference genome. This large unbalanced structural variation can be present in the form of deletions (1 or 0 copies) and duplications (>2 copies). CNVs can cause many rare sporadic and Mendelian disorders, such as split hand/foot malformation and leukodystrophy [1, 2]. Furthermore, severe paediatric conditions are often caused by large CNVs spanning multiple genes, with the size of such CNVs varying from 1 to 3 megabases (Mb) (e.g., DiGeorge and Charcot-Marie-Tooth syndromes [3, 4]) to >10 Mb (e.g., Cri-du-Chat and cat-eye syndromes [5, 6]).

Tens of computer programs for CNV detection have been developed over the last decade, with most being based on read-depth, split-read, read-pair and *de novo* assembly approaches [7, 8]. However, the vast majority of benchmarking analyses tend to focus on relatively small variants rather than on big, clinically actionable alterations in the genome [9–12] (see [13] for an example of a study showing the performance of one popular CNV caller on CNVs >1 Mb). Consequently, there are no comprehensive benchmarks assessing the performance of different tools on large deletions and duplications, a situation which can be partly explained by the limited availability of samples with genetic aberrations of this kind.

We present ConanVarvar, a novel software for quick and robust joint calling of large, syndromic CNVs in batches of whole-genome sequencing (WGS) samples using read depth. To aid in the analysis, ConanVarvar annotates identified CNVs with information about associated syndromic conditions and generates plots showing the position of each variant on the chromosome. ConanVarvar demonstrated a superior performance on our test dataset comprising both clinical and simulated samples with large CNVs, compared to some of the most popular programs for CNV analysis.

## Implementation

### Overview of the approach

**Fig. 1:**
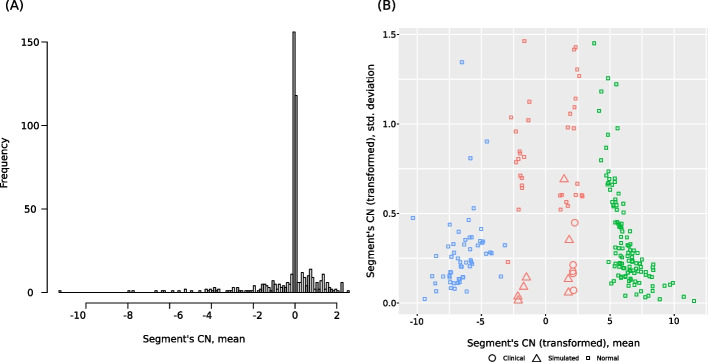
Results of the transformation of all copy number segments from the entire dataset (13 clinical and simulated CNVs). The transformation procedure is used to change the scale of $\log _2$ copy number values for subsequent removal of all non-CNV segments using a *K*-means clustering algorithm. **A** Distribution of segments’ mean values before the transformation. In this distribution, there is no clear separation between normal segments (i.e., those that correspond to the diploid state), which form a peak around zero, and potential CNVs (tails of the distribution). **B** Segments’ mean and standard deviation values after the transformation. Circles and triangles denote segments corresponding to real CNVs from the dataset. After applying clustering on the transformed values, it becomes easy to remove false positives (the blue and green clusters) while keeping all true-positive CNVs (the red cluster, centered around 0)

ConanVarvar is a read depth-based CNV caller with both a graphical user interface (GUI) and a command-line interface (CLI) (Additional file 1: Figs. S1 and S2). It approximates read depth along chromosomes by splitting them into bins of fixed size (e.g., 50 kb) with subsequent corrections for GC content and mappability. To detect abnormal regions, ConanVarvar performs segmentation of binned genomic intervals and assigns each segment an averaged copy number value. When the total number of available segments is sufficient, the program first removes all outliers with high standard deviation and then transforms the mean copy number of each segment to a different scale, so that potential deletions and duplications are further away from other segments (see Fig. 1); a *K*-means clustering algorithm then groups all transformed segments into “normal” and “CNV” categories. Otherwise, when there are fewer than 30 segments available, which renders clustering inefficient, a simple threshold-based approach is triggered to identify abnormal regions based on the raw read depth of the segment.

**Fig. 2:**
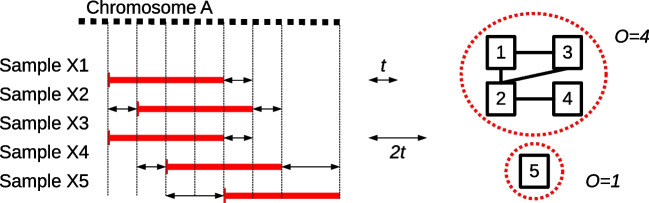
Example of the occurrence calculation. ConanVarvar first calculates the Manhattan distance (in bins) between all identified CNVs and creates an adjacency matrix, which is then used to build a graph. Each disconnected component in the graph corresponds to a group of CNVs that are sufficiently close to each other. The occurrence value is then calculated as the number of nodes in each disconnected component

Once all non-CNV segments are excluded from the list, the program assigns each variant an “occurrence” value indicating the total number of identical CNVs found in other samples using a distance-based estimation technique (see Fig. 2). Finally, ConanVarvar generates plots (Additional file 1: Fig. S3) and reports the identified CNVs, with all per-variant statistics and annotations, including bootstrapped *p*-values, summarised in the form of a spreadsheet (Additional file 1: Fig. S4). A more detailed description of the developed methodology is available in Additional file 1: Note S1, illustrated with the workflow shown in Additional file 1: Fig. S5.

### Benchmarking

#### Tools

We benchmarked ConanVarvar against two commonly used read depth-based tools, CNVnator v0.4 [14] and Control-FREEC v11.5 [15, 16]. The tools were selected based on their superior performance among other similar programs in previous benchmarks, especially on large deletions and duplications [9]. We also included in the comparison an alternative Python implementation of the CNVnator algorithm called CNVpytor (v1.2.1), which provides speed improvements and additional features when compared to its predecessor [17]. Another popular tool, Manta v1.6.0 [18], which is considered one of the top-performing CNV detection algorithms overall in terms of recall, accuracy and precision on both simulated and real data [9, 11], was also added to the comparison. In contrast to ConanVarvar, Control-FREEC and CNVnator/CNVpytor, Manta uses read-pair and split-read information instead of read depth.

#### Data

Due to the extremely deleterious nature of large CNVs, they are much rarer genomic events than their smaller counterparts. For this reason, there is a very limited number of publicly available WGS samples with large CNVs, which results in the lack of benchmarking studies involving this type of variation [9, 10].

**Table 1:** Dataset for benchmarking, containing 14 BAM files with BWA-aligned -150 bp Illumina reads and 25–30$\times$ coverage

Sample	Related to	Source	CNVs of interest	BAM file type
1	–	NA12878 from 1000G Project	None	Full
2	3	Clinical	Deletion in chromosome 10	Full
3	2	Clinical	Deletion in chromosome 10	Full
4	–	Clinical	Two part-deletions in chromosome 5 (5q13.3–14.1 and 5q14.3)	Full
5	–	Clinical	Deletion in chromosome 22 (22q11, DiGeorge syndrome)	Full
6	–	Simulated (EAGLE)	$\sim$1.4 Mb duplication in chromosome 17 (Charcot-Marie-Tooth syndrome type 1A)	Single-chromosome (17)
7	–	Simulated (EAGLE)	8p23.1 ($\sim$3.7 Mb) duplication	Single-chromosome (8)
8	–	Simulated (EAGLE)	16p11.2–12.2 ($\sim$7.8 Mb) duplication	Single-chromosome (16)
9	–	Simulated (EAGLE)	15q26 ($\sim$3.2 Mb) duplication	Single-chromosome (15)
10	–	Simulated (EAGLE)	None	Single-chromosome (22)
11	–	Simulated (BAMSurgeon)	10q22.3–23.2 ($\sim$7.5 Mb) deletion (same deletion as in samples #2 and #3)	Single-chromosome (10)
12	–	Simulated (BAMSurgeon)	5p15.2–15.33 ($\sim$11.3 Mb) deletion (Cri-du-chat syndrome)	Single-chromosome (5)
13	–	Simulated (BAMSurgeon)	16p11.2–12.2 ($\sim$8.7 Mb) deletion	Single-chromosome (16)
14	–	Simulated (BAMSurgeon)	2q33.1 ($\sim$8.3 Mb) deletion	Single-chromosome (2)

To assess the performance of ConanVarvar, CNVnator/CNVpytor, Control-FREEC and Manta, we created a test dataset of 14 WGS files, comprising 4 clinical samples, 9 simulated single-chromosome samples and the original sample NA12878 from the 1000 Genomes Project [19] dataset (see Table 1). The clinical samples were selected based on WGS and comparative genomic hybridisation (CGH) microarray results in cases from our in-house cohort of patients [20]. The dataset contained a total of 13 large (>1 Mb) deletions and duplications. All simulated files were generated using either BAMSurgeon v1.2 [21] or Illumina’s EAGLE simulator v2.5.1 [22] (see Additional file 1: Note S2).

#### Parameters

All read depth-based tools (ConanVarvar, CNVnator/CNVpytor, Control-FREEC) were run at the 50 kb resolution. For ConanVarvar and CNVnator/CNVpytor, the default settings were used. The parameters of Control-FREEC were selected to most closely match the default settings of ConanVarvar, e.g., both programs were tested with the minimum mappability of 0.8. For other parameters, either recommended or default values were used (see Additional file 1: Note S2). For CNVnator, Control-FREEC and Manta, the same reference files were used in each run, either as separate chromosomes (CNVnator and Control-FREEC) or in a merged form (Manta). Allosomes (sex chromosomes) were excluded from the analysis in all samples, as were Manta’s BND (‘breakend’) type of records, specific to inversions and translocations.

#### Evaluation metrics

The performance of ConanVarvar, CNVnator/CNVpytor, Control-FREEC and Manta was evaluated using the F1, precision and recall metrics. The execution time on an HPC (high-performance computing) server node with 4 Intel Xeon CPUs and 128 GB of RAM was recorded for each tool.

## Results

### F1, precision and recall

**Fig. 3:**
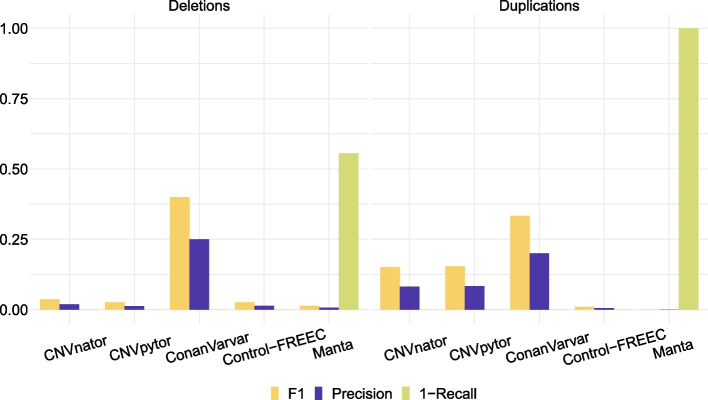
Overall performance of ConanVarvar, CNVnator/CNVpytor, Control-FREEC and Manta on the test dataset based on the merged output of each tool for NA12878 and the 4 clinical and 9 simulated single-chromosome samples. A CNV was considered a false positive if it was not present in the simulated or clinical samples. “1-Recall” is also known as False Negative Rate (FNR). Absence of a bar indicates that the corresponding metric has the value of zero, e.g., ConanVarvar, CNVnator/CNVpytor and Control-FREEC all had the FNR of zero (perfect recall). Manta’s output was filtered to include only those variants that were at least 50 kb in size

Among the tools we evaluated, ConanVarvar had the highest F1 and precision scores, as shown in Fig. 3. It correctly identified all CNVs of interest and reported the smallest number of false positives (i.e., variants other than the selected 13 CNVs). In contrast, Manta had the lowest precision overall. It missed more than half of all CNVs and performed especially poorly on duplications. Interestingly, the performance of Manta on simulated data was better than on real data, where it failed to find most large CNVs (Additional file 1: Figs S6–S11), except for one clinical sample, for which it gave a partially correct answer (compare Additional file 1: Fig. S12 with Figs. S13 and S14). Perhaps unsurprisingly, the output of CNVnator and CNVpytor for most of the samples in our benchmark was identical or nearly identical, which is also reflected in the highly similar F1, precision and recall characteristics of the two tools in Fig. 3.

### Concordance

**Fig. 4:**
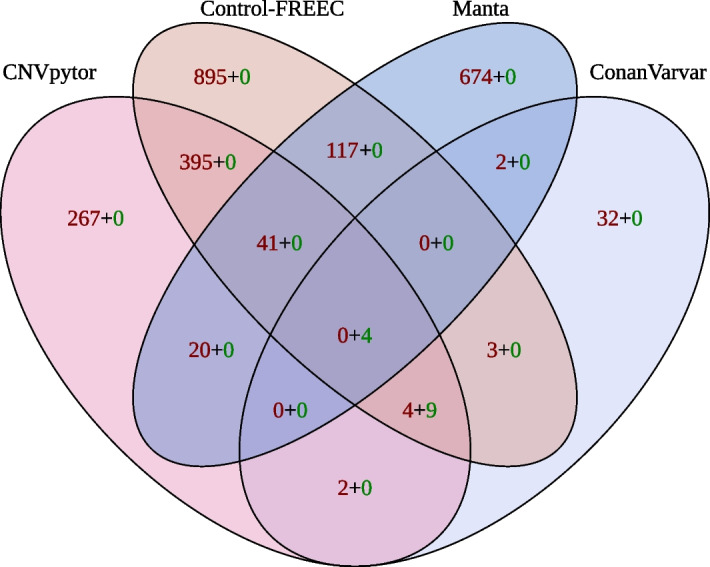
Concordance between ConanVarvar, CNVnator, Control-FREEC and Manta on the test dataset. Colour indicates the total number of false positives (red) and true positives (green). Only 4 variants were correctly identified by all tools. The remaining 9 true positives were reported only by ConanVarvar, CNVnator and Control-FREEC. Variants smaller than 50 kb in Manta’s output were excluded. As CNVnator and CNVpytor are different implementations of the same algorithm and have almost identical performance in terms of true and false positives, only CNVpytor’s calls are shown

As shown in Fig. 4, the overall concordance between the tools was strikingly low. Each tool, with the exception of ConanVarvar, produced a considerably large list of unique false positives. The most obvious examples of such false positives, identified by Manta, are shown in Additional file 1: Figs. S15–S20. Also, we found that both CNVnator/CNVpytor and Control-FREEC treat gaps in the centromere regions as CNVs, which resulted in another group of false-positive calls (Additional file 1: Figs. S21–S29).

### Execution time

#### Single files

**Table 2:** Comparison of computational performance

Tool	Built-in parallelisation enabled?	Simulated samples (single-chromosome BAM files), minutes	Clinical samples (full BAM files), minutes	NA12878 (full BAM file), minutes	Joint calling, minutes
Manta	Auto	2.3(1.7)	76.5(21.0)	70	$397^{\dagger }$
CNVnator	Auto	1.4(0.7)	22.0(0.8)	32	$133^{\dagger }$
CNVpytor	Yes	3.6(2.1)	15.3(0.5)	27	$121^{\dagger }$
Control-FREEC	Yes	2.3(1.2)	31.2(7.2)	36	$182^{\dagger }$
ConanVarvar	Yes	1.7(0.9)	21.5(0.6)	32	63
ConanVarvar	No	1.8(0.8)	22.5(1.3)	33	131

Mean and standard deviation (in brackets) of each tool’s execution time on different BAM files, calculated as the time it takes for a program to output the results of variant calling without running any additional scripts (e.g., for plots). An HPC server node with a total of 128 GB of RAM and 4 Intel Xeon CPUs was used in each run. Symbol $^{\dagger}$ indicates that the value is an estimate

CNVnator and ConanVarvar demonstrated a very similar performance in terms of their execution time on the analysed samples (Table 2). Control-FREEC was slightly slower on full BAM files compared to ConanVarvar and CNVnator, and was on average as fast as the other two programs on single-chromosome samples. Manta was the slowest of the five tools on full BAMs, which, however, can be explained by the underlying differences between split-read, read-pair and read depth-based methods. In particular, it demonstrated a two- to three-fold slower performance compared to ConanVarvar, CNVnator and Control-FREEC on each of the full BAM files, though its performance on single-chromosome samples was not too inferior. Interestingly, even though CNVpytor was significantly faster on full BAM files than the other four tools, it was surprisingly slow on single-chromosome BAMs.

#### Joint calling

None of the above-mentioned tools, except for ConanVarvar, allow for concurrent processing of multiple WGS samples. Even though Manta can, in principle, be run on several BAM files simultaneously, it is reported to fail on datasets with more than 12 samples, and, therefore, this feature was not applicable to our dataset. Hence, apart from ConanVarvar, all other tools required multiple invocations to analyse files from our dataset.

ConanVarvar is different, as it was specifically designed for quick multi-sample analysis. As shown in Table 2, it batch-processed the entire dataset in just 63 min, efficiently utilising all available CPUs (configurable behaviour).

### Prioritisation

When ConanVarvar was run on all 14 BAM files as one large batch of samples, it reported only 50 variants in total, a quantity 10–30 times smaller than the total number of false-positive CNVs outputted by the other four tools. Importantly, not only did it find all real CNVs of interest, but it also prioritised those variants based on their calculated *p* values and associated syndromes, such that all of them were in the first half of the list. Besides, in order to aid in further filtering of false positives, ConanVarvar calculated within-batch “occurrence” values for all identified variants based on the nearness of each one of them to other variants.

## Discussion

CNVs larger than 1 Mb remain a considerably understudied class of genomic variants in bioinformatics [9–12]. Yet, these variants cause some of the most severe developmental disorders and should, therefore, be prioritised. In this paper we present ConanVarvar, a robust CNV detection tool based on read depth that specifically addresses the problem of large deletions and duplications. This software is designed to be used as a primary analysis program for simultaneous screening of multiple WGS samples for the most deleterious mutations. The 1 Mb cutoff on the CNV size allows ConanVarvar to quickly detect tens of known syndromic CNVs without reporting large numbers of false positives. False-positive CNVs are a long-known problem in disease sequencing studies [7, 23, 24], as they tend to complicate the analysis by obscuring the more severe genomic abnormalities.

Our benchmarking results show that read depth-based CNV callers, such as ConanVarvar, CNVnator/CNVpytor and Control-FREEC, tend to perform well on large CNVs. In contrast, despite being one the *de facto* best algorithms for the detection of structural variation according to previous studies, Manta, which utilises split-read and read-pair information for CNV detection, missed half of all the large CNVs in our dataset. In particular, it did extremely poorly on our large duplications, which is strikingly different to its previously reported performance on CNVs of this type with smaller size [9]. Among the read depth-based callers in this study, ConanVarvar demonstrated superior results in terms of the F1 and precision metrics on both real and simulated data by making several-fold less false-positive calls and prioritising true positives higher. Consistent with the literature [13], CNVnator correctly identified all large CNVs from our dataset, as did Control-FREEC. Nevertheless, the overall concordance between the tools was rather low, with the vast majority of calls being unique false positives.

Aside from the superior performance, ConanVarvar offers built-in annotations, facilitating the identification of clinically actionable CNVs based on 56 known syndromes. As WGS technology is gradually becoming a standard clinical practice, we anticipate that the reliance on such readily available annotation features will increase accordingly in the near future.

One of the limitations of our study is the size of our selection of CNV callers. Besides, we were also limited in the number of metrics we could use for the benchmarking, as the definition of a true negative is ambiguous in the context of large CNVs. However, given that our test dataset was sufficiently heterogeneous, we argue that the above-mentioned conclusions are still valid.

In future developments of ConanVarvar, we plan to add the support of single-nucleotide variants integration, in order to improve the accuracy of CNV breakpoint detection, which is currently one of the major limitations of all programs based on read depth, as the accuracy depends on the bin size. We also plan to regularly update the list of syndromes which is used for annotations in ConanVarvar.

## Conclusions

We believe that this work will not only provide the bioinformatics community with a new tool for CNV analysis but will also help to further elucidate the performance of existing tools for CNV detection on large CNVs.

## Supplementary Information

**Additional file 1**. File contains supplementary information about the methods (**Note S1**) and the benchmarking procedure (**Note S2**). **Fig. S1**. The graphical user interface (GUI) of ConanVarvar, developed using the R Shiny framework. **Fig. S2**. The command-line interface (CLI) of ConanVarvar. **Fig. S3**. Examples of plots generated by ConanVarvar. **Fig. S4**. Example of the produced spreadsheet with pre-sorted candidate variants. **Fig. S5**. Complete workflow diagram of ConanVarvar. **Figs. S6–S29**. Sample plots created using ConanVarvar's native plotting function showing the output of Manta, CNVnator and Control-FREEC for some of the samples used in the benchmarking.

## Data Availability

The source code and test data are available online at https://github.com/VCCRI/ConanVarvar. The datasets generated and/or analysed during the current study are not publicly available due to the use of patient-identifiable clinical data, but are available from the corresponding author on reasonable request. Project name: ConanVarvar; Project home page: https://github.com/VCCRI/ConanVarvar; Docker Hub: https://hub.docker.com/r/mgud/conanvarvar; Operating systems: Platform independent; Programming languages: R; Other requirements: Docker; License: GNU GPL.

## Abbreviations

CGH: Comparative genomic hybridisation
CHD: Congenital heart disease
CLI: Command-line interface
CNV: Copy number variant
FNR: False negative rate
GUI: Graphical user interface
HPC: High-performance computing
Mb: Megabase
WGS: Whole-genome sequencing
kb: Kilobase
