# Supplementary material for: ConanVarvar: a versatile tool for the detection of large syndromic copy number variation from whole-genome sequencing data
Source: BMC Bioinformatics. 2023 Feb 15;24:49. doi: 10.1186/s12859-023-05154-x (PMC9930243; doi:10.1186/s12859-023-05154-x)
Supplement: Supplementary file 1 — Additional file 1. File contains supplementary information about the methods (Note S1) and the benchmarking procedure (Note S2). Fig. S1. The graphical user interface (GUI) of ConanVarvar, developed using the R Shiny framework. Fig. S2. The command-line interface (CLI) of ConanVarvar. Fig. S3. Examples of plots generated by ConanVarvar. Fig. S4. Example of the produced spreadsheet with pre-sorted candidate variants. Fig. S5. Complete workflow diagram of ConanVarvar. Figs. S6–S29. Sample plots created using ConanVarvar's native plotting function showing the output of Manta, CNVnator and Control-FREEC for some of the samples used in the benchmarking. [file 12859_2023_5154_MOESM1_ESM.pdf]

Supplementary Materials

Contents

Supplementary Note 1: Methods **2**
Technical specifications 2
Workflow 2
GC and Mappability correction 2
Segmentation 3
Post-segmentational processing 3
List of syndromes that are automatically identified by ConanVarvar 4

Supplementary Note 2: Benchmarking **5**
Data simulation approach 5
Coordinates of the simulated CNVs 5
Manta 6
Control-FREEC 6
Configuration files 6
"bam_info" file for NA12878 6

Supplementary Figures **7**

Supplementary Note 1: Methods

Technical specifications

ConanVarvar is a Docker program, which can be run in the GUI mode (Figure S1) or in the command-line mode (Figure S2). The program takes Binary Alignment Map (BAM) files as the input and outputs two types of data: per-chromosome PDF plots (optional) and a spreadsheet with identified variants. Some regions in the plots are coloured to help the user filter out false positives, which is particularly important when working with read depth data, which tends to be quite noisy. Entries in the spreadsheet are presorted in the decreasing order of their significance. The user is advised to carefully look through the first few large CNVs with low occurrence, especially the ones that are syndromic, and cross-check these with the plots. Examples are shown in Figure S3 and Figure S4.

In-built multi-level parallelisation makes the tool faster than analogous programs, especially when run on large batches of multiple samples. Importantly, the tool does not require reference FASTA files, thereby facilitating reproducibility of CNV analysis. Other features include on-the-fly sorting and indexing of the input BAM files, filtering of false positives based on the proximity to the centromere and overlaps with segmental duplications and reporting of the occurrence of each CNV. Besides, ConanVarvar also annotates identified variants, should they have overlaps with any of the known syndromic CNVs from the DECIPHER database [1]. The two databases provide information about known genetic abnormalities and their relationship to various phenotypes. Hence, this information can be utilised to flag and prioritise CNVs that have been shown to be associated with certain syndromes.

Workflow

The complete workflow of the program includes the following major steps (also shown in Figure S5):

1. Sort and index the input files if required;
2. Count reads that fall into preconfigured genomic bins of fixed size;
3. Correct the read counts for biases relating to GC content and mappability and convert the counts to scaled copy number values [2]–[4];
4. Perform segmentation on the copy number values using the `fastseg` package [5];
5. Assign a state (normal, deletion, insertion) to each of the obtained segments and calculate per-segment statistics;
6. Perform final filtering and generate plots.

GC and Mappability correction

The following algorithm, based on single-position (*i.e.* single base pair) model estimation, is used for GC and mappability correction in ConanVarvar (adapted from the `HMMcopy` R package) [2], [6]:

1. Filter out bins with 0 reads and 0 GC content;
2. Filter out bins with reads within the top and bottom 1% quantile;
3. Filter out bins with GC content within the top and bottom 1% quantile;
4. Filter out bins with a mappability score above 0.8 (unless changed);
5. Randomly sample the remaining high-quality bins;
6. The first LOESS (on the reads-by-GC curve) with a small span (smoothing window) is performed, obtaining typically a highly sensitive curve (follows low density tails of distribution, but gets jagged in high density center);
7. A second LOESS (on the first LOESS results) with a larger span is performed, recapitulating the curve in the low density tails and smoothing out the jagged regions in the high density center;
8. GC correction: the number of observed reads is divided by the number of reads predicted by the LOESS curve given an observed GC proportion;
9. Filter out the top 1% quantile of the GC-corrected bins, then randomly sample the remaining bins;
10. A separate LOESS curve is computed for mappability-by-GC;
11. Mappability correction: values are divided by the value predicted by the mappability LOESS curve generated in the previous step;
12. Convert to $\log_2$ copy number values.

Segmentation

ConanVarvar uses the `fastseg()` function from the `fastseg` R package to segment arrays of chromosome-wide copy number values into contiguous intervals with consistent copy number [5]. The algorithm identifies breakpoints in copy number data based on the cyber *t*-test which makes it much faster than the widely used circular binary segmentation [7]–[9]. To minimise the number of false positives (small segments and segments with high standard deviation), the following empirically-derived parameter values were chosen:

`alpha = 0.01`	Parameter controlling 'the ratio of initial breakpoints'. In other words, the higher the value of `alpha`, the more segments are produced. Since CNVs >1 Mb are very rare genomic events, the value of `alpha` was set to 0.01 (the default is 0.1).
`cyberWeight = 1`	Parameter controlling 'the weight of the global variance'. Since real CNVs generally have relatively low variance, the value of `cyberWeight` was set to 1 to minimise the number of small segments with high variance.
`minSeg = 1e6 / bin.size`	ConanVarvar targets CNVs that are at least 1 Mb in size. Therefore, the value of `minSeg` (minimal segment length) was set to 1 Mb divided by the resolution.

Post-segmentational processing

Clustering After the segmentation step, ConanVarvar classifies the obtained segments into two groups, namely 'CNVs' and 'normal'. If there are too few segments available (< 30), which is often the case in single-chromosome BAM files, a simple threshold-based approach is used to separate potential CNVs from normal segments. By default, ConanVarvar uses –0.7 for deletions and +0.5 for duplications.

Otherwise, if the number of segments is sufficient, ConanVarvar uses a transformation-plus-clustering procedure. It first removes all outliers with high standard deviation, and then rescales each segment's mean copy number using the following formula:

$$\mu' = \text{sign}(\mu) * \log\left(\frac{|\mu|}{\max(|\mu|)}\right)$$

After that, ConanVarvar runs `stats::kmeans()` on the transformed mean values with centers in $\left[\min(\mu'), 0, \max(\mu')\right]$. This function creates three clusters, of which only the middle one (centered around $\mu' = 0$) contains potential CNVs. The original value of μ is then used to separate deletions from duplications in that cluster.

Bootstrap Once all normal segments are excluded from the analysis, ConanVarvar calculates one-sided *p*-values separately for deletions and duplications using the following method:

1. For each observed segment length, create a null distribution of copy number values:
    1. Add the copy number of all observed segments with the given length to a vector;
    2. Create artificial segments by sampling with replacement from all available bins, so that the total number of segments (both real and artificial) is 1000;
    3. Sort the obtained values.
2. Using the obtained bootstrap distributions, calculate one-sided *p*-values for each deletion or duplication.

Occurrence It is common that due to noise in read-depth data there can be false positives that are common across the whole batch. To account for this, ConanVarvar first creates an adjacency matrix for all potential CNVs it has identified, which is based on how far each CNV is from other CNVs. This distance is calculated using a variation of the Manhattan distance metric based on the number of bins between start and end positions of every two CNVs. After that, the occurrence value is assigned to all CNVs that form a group. That is, all unique CNVs have the occurrence of 1.

List of syndromes that are automatically identified by ConanVarvar

ConanVarvar can identify a total of 56 syndromes, of which most were taken from the DECIPHER database of CNV-associated syndromic conditions. Out of those 56, 38 are larger than 1 Mb. At the time of writing, the database contained 54 autosomal and 12 were allosomal (sex-linked) CNVs. The 2 conditions that were not present in DECIPHER (the 10q22.3–23.2 deletion syndrome and the 12p13.33 microdeletion syndrome) were added from the OMIM database, based on the manual analysis of our in-house CHD samples.

```
1p36 microdeletion syndrome
1q21.1 susceptibility locus for Thrombocytopenia-Absent Radius (TAR) syndrome
1q21.1 recurrent microdeletion (susceptibility locus for neurodevelopmental disorders)
1q21.1 recurrent microduplication (possible susceptibility locus for neurodevelopmental disorders)
2p21 Microdeletion Syndrome
2p15-16.1 microdeletion syndrome
2q33.1 deletion syndrome
2q37 monosomy
3q29 microdeletion syndrome
3q29 microduplication syndrome
Wolf-Hirschhorn Syndrome
Cri du Chat Syndrome (5p deletion)
Familial Adenomatous Polyposis
Adult-onset autosomal dominant leukodystrophy (ADLD)
Sotos syndrome
Williams-Beuren Syndrome (WBS)
7q11.23 duplication syndrome
Split hand/foot malformation 1 (SHFM1)
8p23.1 duplication syndrome
8p23.1 deletion syndrome
8q21.11 Microdeletion Syndrome
9q subtelomeric deletion syndrome
10q22.3-23.2 deletion
WAGR 11p13 deletion syndrome
Potocki-Shaffer syndrome
12p13.33 Microdeletion Syndrome
12q14 microdeletion syndrome
Angelman syndrome (Type 1)
Prader-Willi syndrome (Type 1)
Prader-Willi Syndrome (Type 2)
Angelman syndrome (Type 2)
15q13.3 microdeletion syndrome
15q24 recurrent microdeletion syndrome
15q26 overgrowth syndrome
ATR-16 syndrome
Rubinstein-Taybi Syndrome
16p13.11 recurrent microdeletion (neurocognitive disorder susceptibility locus)
16p13.11 recurrent microduplication (neurocognitive disorder susceptibility locus)
16p11.2-p12.2 microduplication syndrome
16p11.2-p12.2 microdeletion syndrome
Recurrent 16p12.1 microdeletion (neurodevelopmental susceptibility locus)
16p11.2 microduplication syndrome
Miller-Dieker syndrome (MDS)
Charcot-Marie-Tooth syndrome type 1A (CMT1A)
Hereditary Liability to Pressure Palsies (HNPP)
Smith-Magenis Syndrome
Potocki-Lupski syndrome (17p11.2 duplication syndrome)
NF1-microdeletion syndrome
RCAD (renal cysts and diabetes)
17q21.31 recurrent microdeletion syndrome (Koolen de Vries syndrome)
Early-onset Alzheimer disease with cerebral amyloid angiopathy
Cat-Eye Syndrome (Type I)
22q11 deletion syndrome (Velocardiofacial / DiGeorge syndrome)
22q11 duplication syndrome
22q11.2 distal deletion syndrome
22q13 deletion syndrome (Phelan-Mcdermid syndrome)
```

Supplementary Note 2: Benchmarking

Data simulation approach

We used BAMSurgeon v1.2 [10] and Illumina's EAGLE v2.5.1 [11] to simulate large CNVs in 8 single-chromosome samples.
For deletions, we used BAMSurgeon. The procedure was as follows:

1. Extract the reads for the target chromosome from the BAM file of NA12878 into a new BAM and index the new file with Samtools

2. Simulate the CNV of interest

```
python /path/to/bamsurgeon/bin/addsv.py \
        --varfile /path/to/varfile.txt \
                --n 1 --procs 2 \
                        --bamfile /path/to/single_chromosome_NA12878.bam \
                                --reference /path/to/hg38.fa \
                                        --outbam simulated.bam \
                                                --maxlibsize 100
```

For duplications, we used EAGLE. The procedure was as follows:

1. Extract per-chromosome reference files from the main FASTA file into `chromosomes/`

```
awk '$0 ~ "^>" {match($1,/^>([^:]+)/,id);filename=id[1]} {print >> filename".fa"}' hg38.fa
rm *v1.fa
rm *v2.fa
rm *decoy.fa
rm HLA*.fa
rm *random.fa
rm *alt.fa
rm GRCh38*.fa
```

2. Start EAGLE

```
docker pull ljanin/eagle:2.5.1
docker run \
       -it --rm \
       --mount type=bind,source=/home/ubuntu/chromosomes,target=/chromosomes \
       ljanin/eagle:2.5.1
```

3. Create a TAB file for the target chromosome with the coordinates of the CNV

4. Generate a VCF for the target chromosome (example)

```
/usr/local/libexec/EAGLE/applyCopyNumber.pl \
    --input-cnv=CNchr17.tab \
    --output-vcf=CNchr17.vcf \
    --default-ploidy=2
```

5. Add the CNV (example)

```
/usr/local/bin/configureEAGLE.pl \
    --reference-genome=/chromosomes/chr17.fa \
    --variant-list=/chromosomes/CNchr17.vcf \
    /chromosomes/EAGLE_chr17
cd /chromosomes/EAGLE_chr17
make -j 4 && make bam
```

The final step in both cases was to check the header, sort and index the new file with Samtools.

Coordinates of the simulated CNVs

`varfile` files for BAMSurgeon with coordinates taken either directly from DECIPHER or from clinical studies (merged):

```
#CHR FIRST_POS LAST_POS TYPE REDUCTION_IN_COPY_NUMBER
chr2 196060397 204342216 BIGDEL 0.4
chr16 21500741 30188533 BIGDEL 0.4
chr10 79689360 87223773 BIGDEL 0.4
chr5 151621 11411588 BIGDEL 0.5
```

TAB files for EAGLE with coordinates taken from DECIPHER (merged):

```
#CHR FIRST_POS LAST_POS INCREASE_IN_COPY_NUMBER
chr17 14194598 15567589 3
chr16 21463739 29272756 4
chr15 98814741 101981189 5
chr8 8242533 11907120 3
```

Manta

ConanVarvar, CNVnator/CNVpytor and Control-FREEC are based on read depth, whereas Manta uses read-pair and split-read information for CNV calling. Thus, Manta can detect a wider range of SV types, and, unlike the other three programs, its resolution is not limited to the size of one bin. As a result, when Manta was run on our dataset, it outputted thousands of CNVs, including many that were smaller than 50 kb (the resolution that ConanVarvar, CNVnator/CNVpytor and Control-FREEC were run with). Therefore, in order not to understate the performance of Manta relative to other tools in terms of false positives, we filtered out all variants from Manta's `diploidSV.vcf` file that were smaller than 50 kb, as well as all non-CNVs (*i.e.* only `MantaDEL` and `MantaDUP` calls were retained).

Control-FREEC

Configuration files

The following parameters were used in all configuration files for Control-FREEC:

```
ploidy = 2

breakPointThreshold = .8

window = 50000
minMappabilityPerWindow = 0.8

contaminationAdjustment = FALSE
contamination = 0

forceGCcontentNormalization = 2

numberOfProcesses = 4
maxThreads = 4

inputFormat = BAM
mateOrientation = 0
```

"bam_info" file for NA12878

```
Program_Version        v11.5
Sample_Name        NA12878.bam
Control_Used        False
CGcontent_Used        True
Mappability_Used        False
Looking_For_Subclones        False
Breakpoint_Threshold        0.8
Window        50000
Number_Of_Reads|Pairs_In_Sample        1533527944
Number_Of_Reads|Pairs_In_Control        0
Output_Ploidy        2
Sample_Purity        1
Good_Polynomial_Fit        True
```

Supplementary Figures

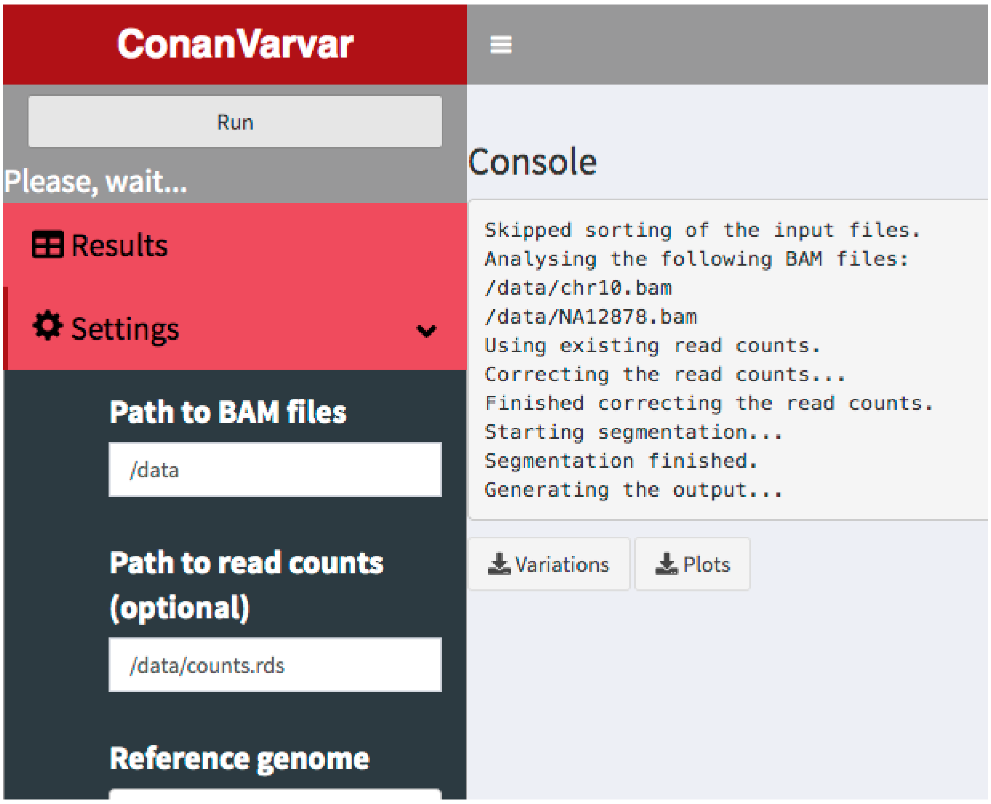

Figure S1: The graphical user interface (GUI) of ConanVarvar, developed using the R Shiny framework. The user has to first specify all the input parameters on the left-hand side of the screen, and then click the 'Run' button, which starts the execution. All intermediate results will appear in the console window.

```
(base) → ~ docker run mgud/conanvarvar --help
Usage: ConanVarvar.R [options]


Options:
	--bamdir=/PATH/TO/DATA
		Full path to the directory with BAM files to be processed

	--counts=/PATH/TO/COUNTS.RDS
		Full path to read counts from previous runs

	--reference=HG38
		Reference genome: hg38 or hg19 [default hg38]

	--format=UCSC
		Format of input sequences: UCSC or NCBI [default UCSC]

	--sortbam
		Sort the input BAM files

	--indexbam
		Index the input BAM files

	--outdir=/OUTPUT
		Output directory

	--binsize=50000
		Bin size [default 50000]

	--minmapq=0.8
		Minimum mapping quality of reads [default 0.8]

	--roughspan=0.1
		First (rough) Loess span [default 0.1]

	--finalspan=0.3
		Second (final) Loess span [default 0.3]

	--delthresh=-0.7
		Threshold for deletions [default -0.7]

	--dupthresh=0.5
		Threshold for duplications [default 0.5]

	--centrmargin=500000
		Centromeres margin [default 500000]

	--segdupthresh=100000
		Lower threshold for segmental duplications [default 100000]

	--occurrencemargin=6
		Start/End margin of error for the Occurrence

	--ncores=4
		Number of cores for parallelisation [default 4]

	--plotresults
		Plot the results

	--verbose
		Print extra output [default]

	-h, --help
		Show this help message and exit
```

Figure S2: The command-line interface (CLI) of ConanVarvar.

(A)

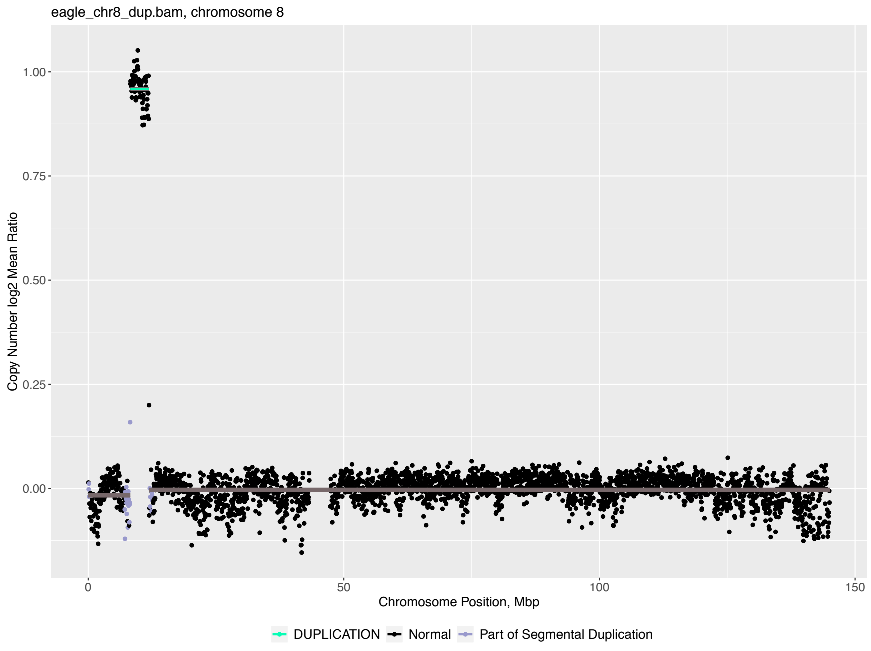

(B)

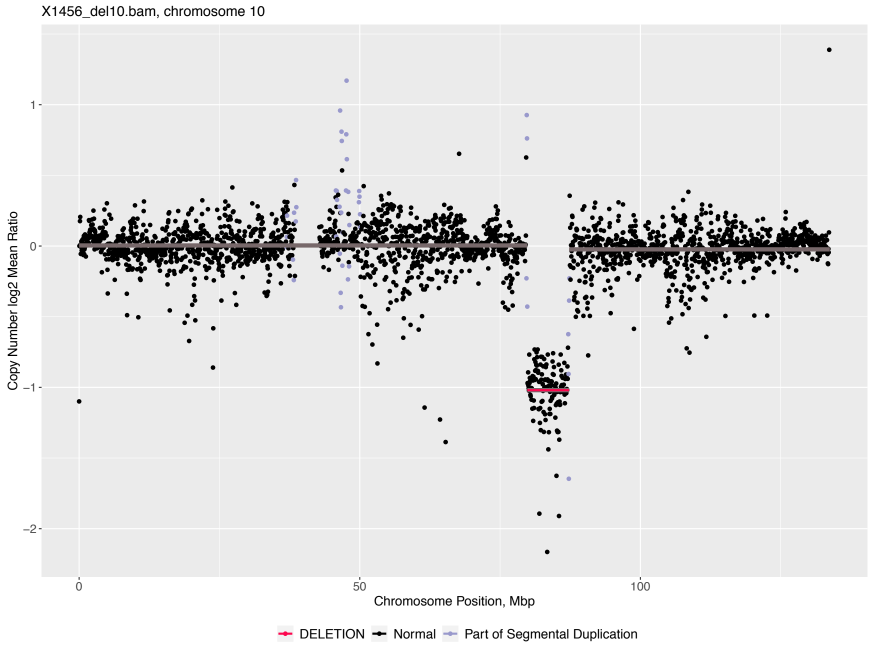

Figure S3: Examples of plots generated by ConanVarvar. Each dot represents an averaged copy number value on the logarithmic scale over a genomic interval determined by the choice of the bin size (here, 50 kb resolution was used). Horizontal lines denote segments, *i.e.* regions of equal copy number. The zero line corresponds to the normal diploid state (when both copies are present). The small gap that is present in both examples at around 40 Mb from the start of the chromosome indicates the centromere. Due to abnormalities in the read depth of regions that are either inside the centromere or sufficiently close to it, ConanVarvar removes all bins that fall into those regions prior to the segmentation step. Segments that have overlaps with segmental duplications larger than a user-defined threshold (purple) are flagged in the final list of potential CNVs. Entries in the output are presorted in the decreasing order of their significance. The user is advised to carefully look through the first few large CNVs with low inter-batch frequency, especially the ones that are syndromic, and cross-check these with the plots. **(A)** Chromosome 8 duplication in a sample simulated using EAGLE. **(B)** Chromosome 10 deletion in a clinical sample.

	A	B	C	D	E	F	G	H	I	J	K
1	Chromosome	Start	End	Width	ID	Copy Number	CNV type	Overlaps with a seg	Occurrence	Associated syndromes	P-value
2	5	1	11400000	11400000	NA12878_CDCS.bam	-1.9585659943	DELETION	No	1	Cri du Chat Syndrome	0.001
3	17	14200001	15550000	1350000	eagle_chr17_dup.bam	0.9839895742	DUPLICATION	No	1	Charcot-Marie-Tooth s	0.001
4	8	8250001	11900000	3650000	eagle_chr8_dup.bam	0.9590406108	DUPLICATION	No	1	8p23.1 duplication syn	0.001
5	8	11650001	12700000	1050000	X1456_del10.bam	1.512449202	DUPLICATION	Yes	1	8p23.1 duplication syn	0.001

Figure S4: Example of the produced spreadsheet with pre-sorted candidate variants.

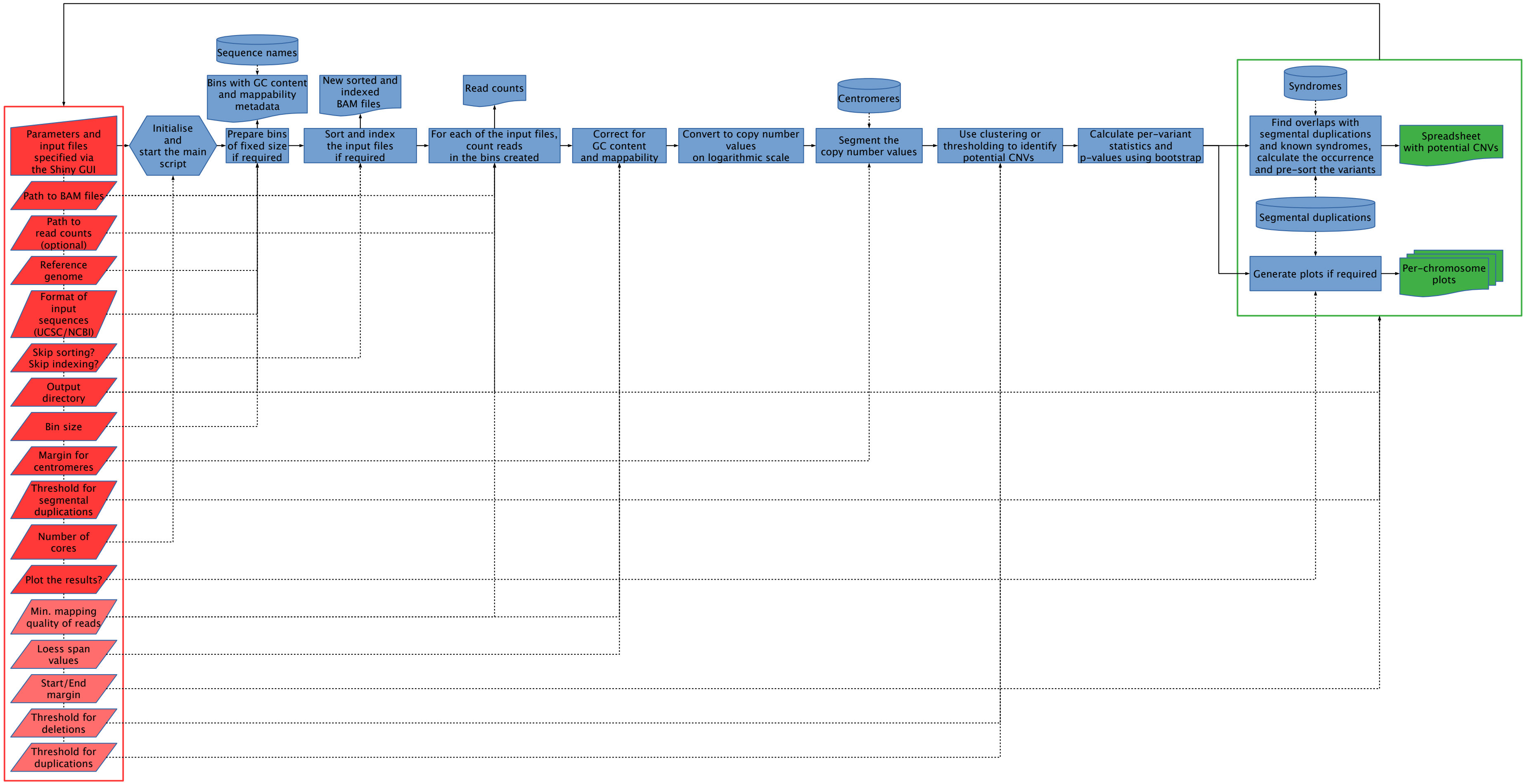

Figure S5: Complete workflow diagram of ConanVarvar. The user starts the program by launching the GUI and specifying the input parameters (red boxes). The 'Run' button in the interface starts the main script of the program, which executes each of the steps of the pipeline in the sequential order (blue boxes). When the execution is finished, the user can collect the generated output files (green boxes) by downloading them via the corresponding buttons in the GUI. The same result can also be achieved by running the program in the command-line mode.

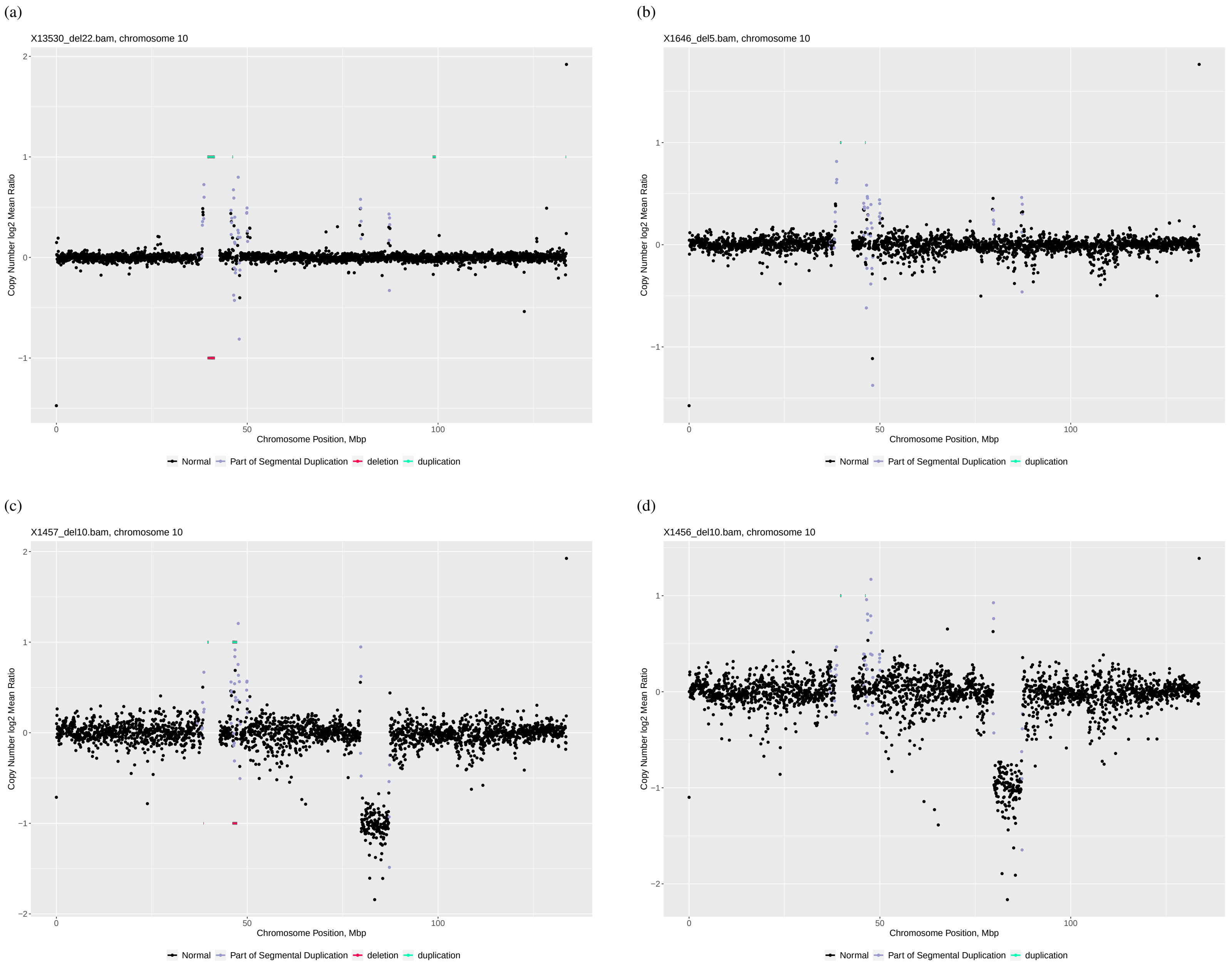

Figure S6: Manta's output for chromosome 10 in 4 clinical samples. The plot was created using ConanVarvar's native plotting function and precomputed copy number values with real calls from Manta. All deletions and duplications were assigned the values of –1 and +1 respectively on the logarithmic copy number scale. As shown in (c) and (d), Manta missed the CNVs of interest.

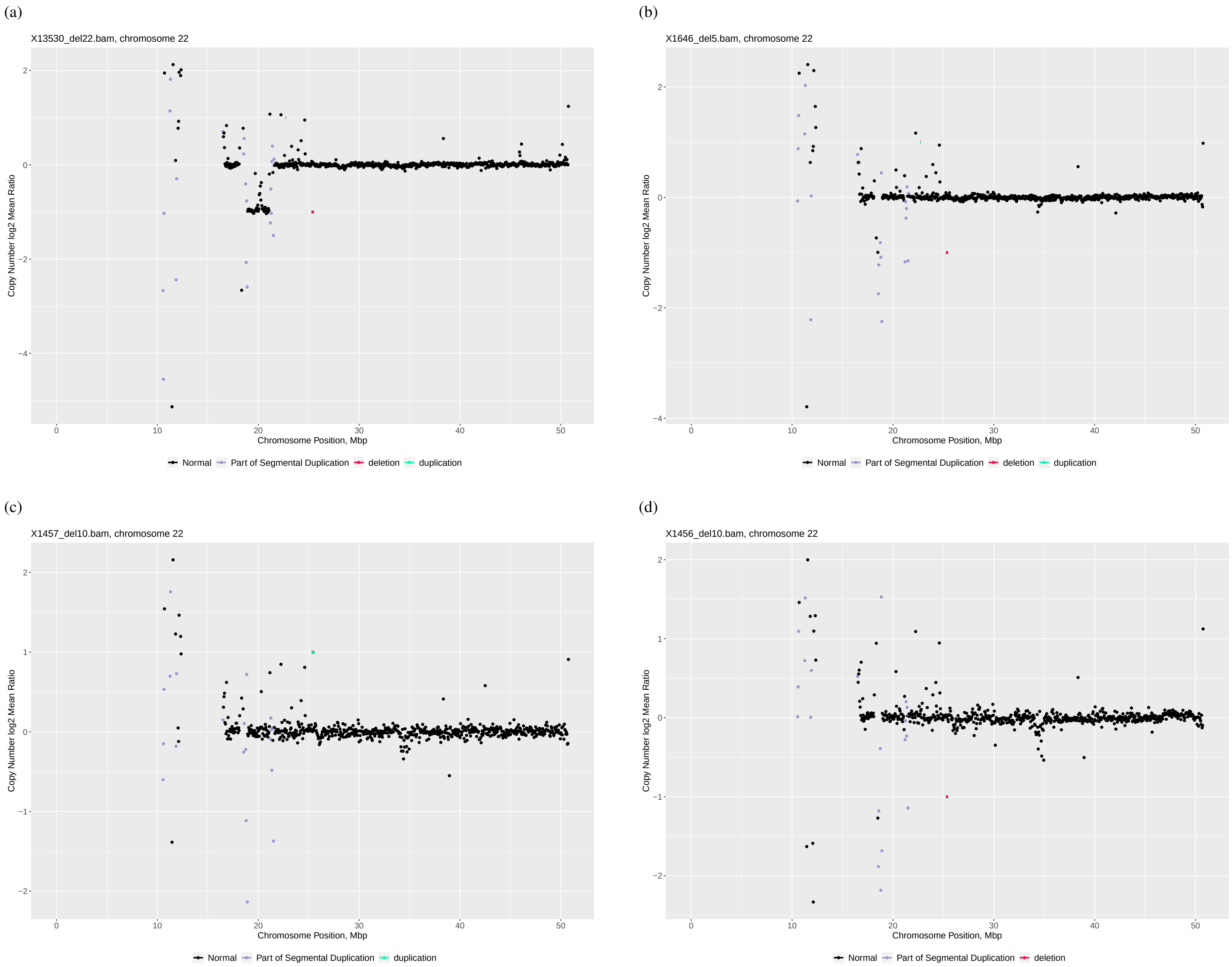

Figure S7: Manta's output for chromosome 22 in 4 clinical samples. The plot was created using ConanVarvar's native plotting function and precomputed copy number values with real calls from Manta. All deletions and duplications were assigned the values of –1 and +1 respectively on the logarithmic copy number scale. As shown in (a), Manta missed the CNV of interest.

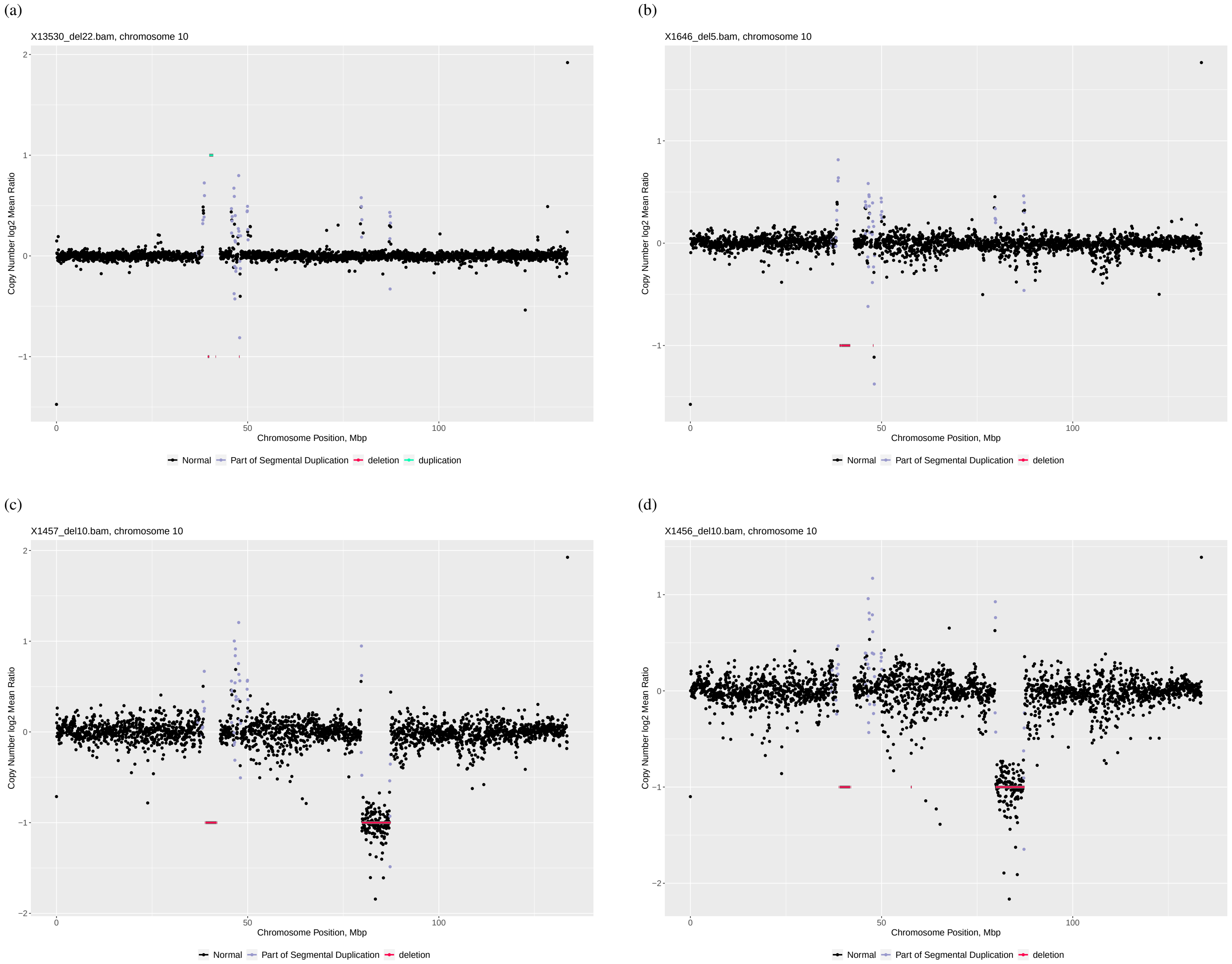

Figure S8: CNVnator's output for chromosome 10 in 4 clinical samples. The plot was created using ConanVarvar's native plotting function and precomputed copy number values with real calls from CNVnator. All deletions and duplications were assigned the values of –1 and +1 respectively on the logarithmic copy number scale. As shown in (c) and (d), CNVnator successfully identified the CNVs of interest.

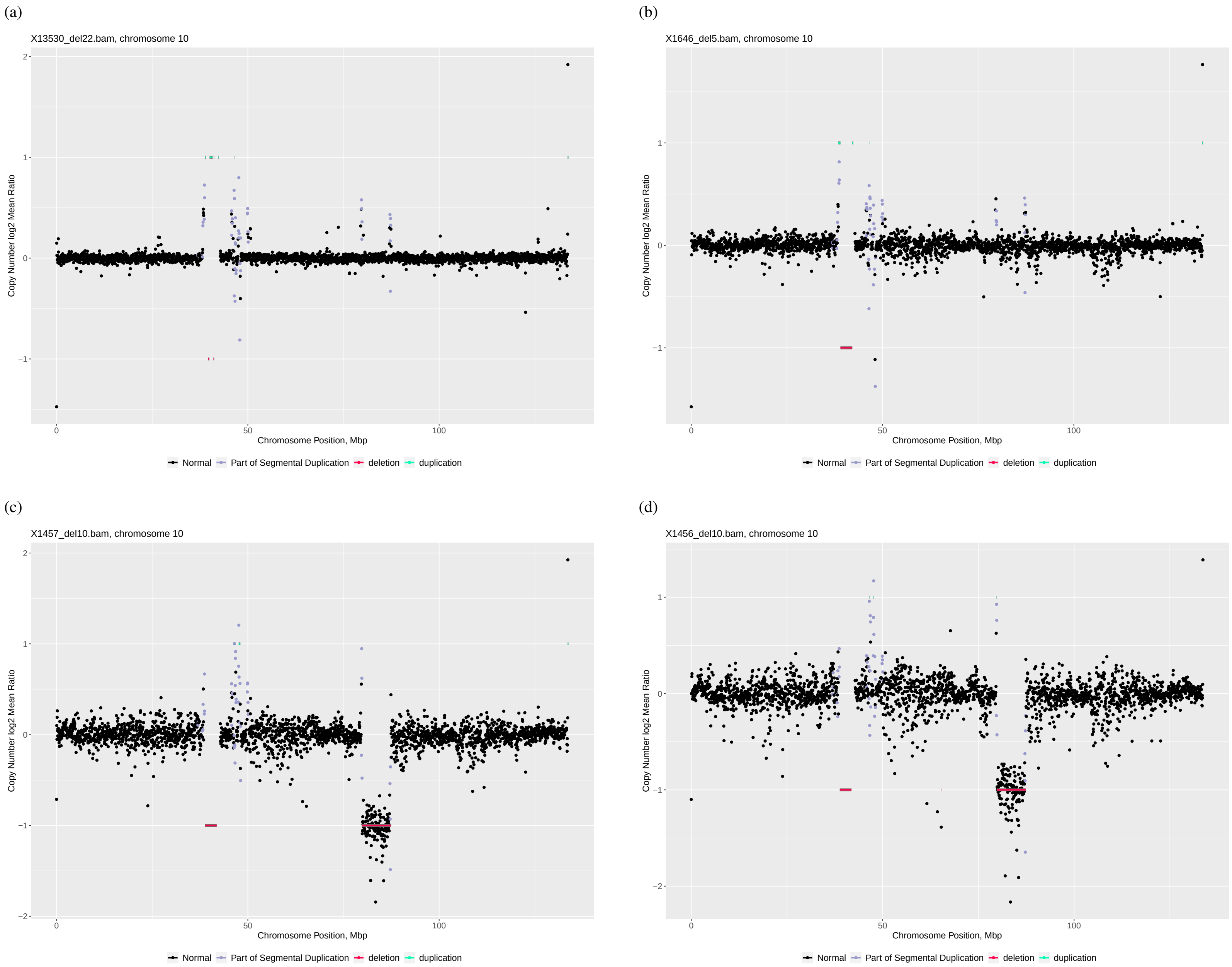

Figure S9: Control-FREEC's output for chromosome 10 in 4 clinical samples. The plot was created using ConanVarvar's native plotting function and precomputed copy number values with real calls from Control-FREEC. All deletions and duplications were assigned the values of –1 and +1 respectively on the logarithmic copy number scale. As shown in (c) and (d), Control-FREEC successfully identified the CNVs of interest.

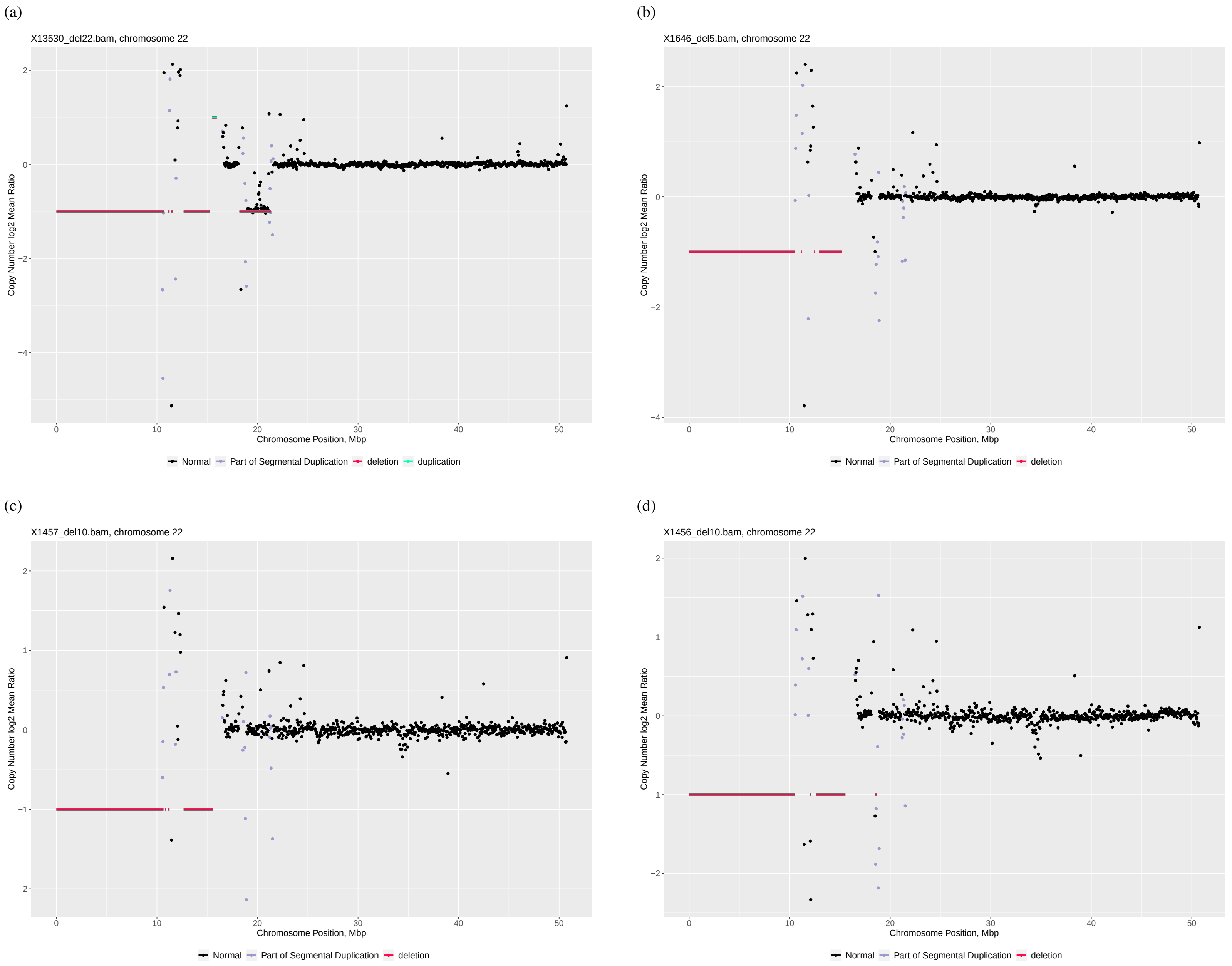

Figure S10: CNVnator's output for chromosome 22 in 4 clinical samples. The plot was created using ConanVarvar's native plotting function and precomputed copy number values with real calls from CNVnator. All deletions and duplications were assigned the values of –1 and +1 respectively on the logarithmic copy number scale. As shown in (a), CNVnator successfully identified the CNV of interest.

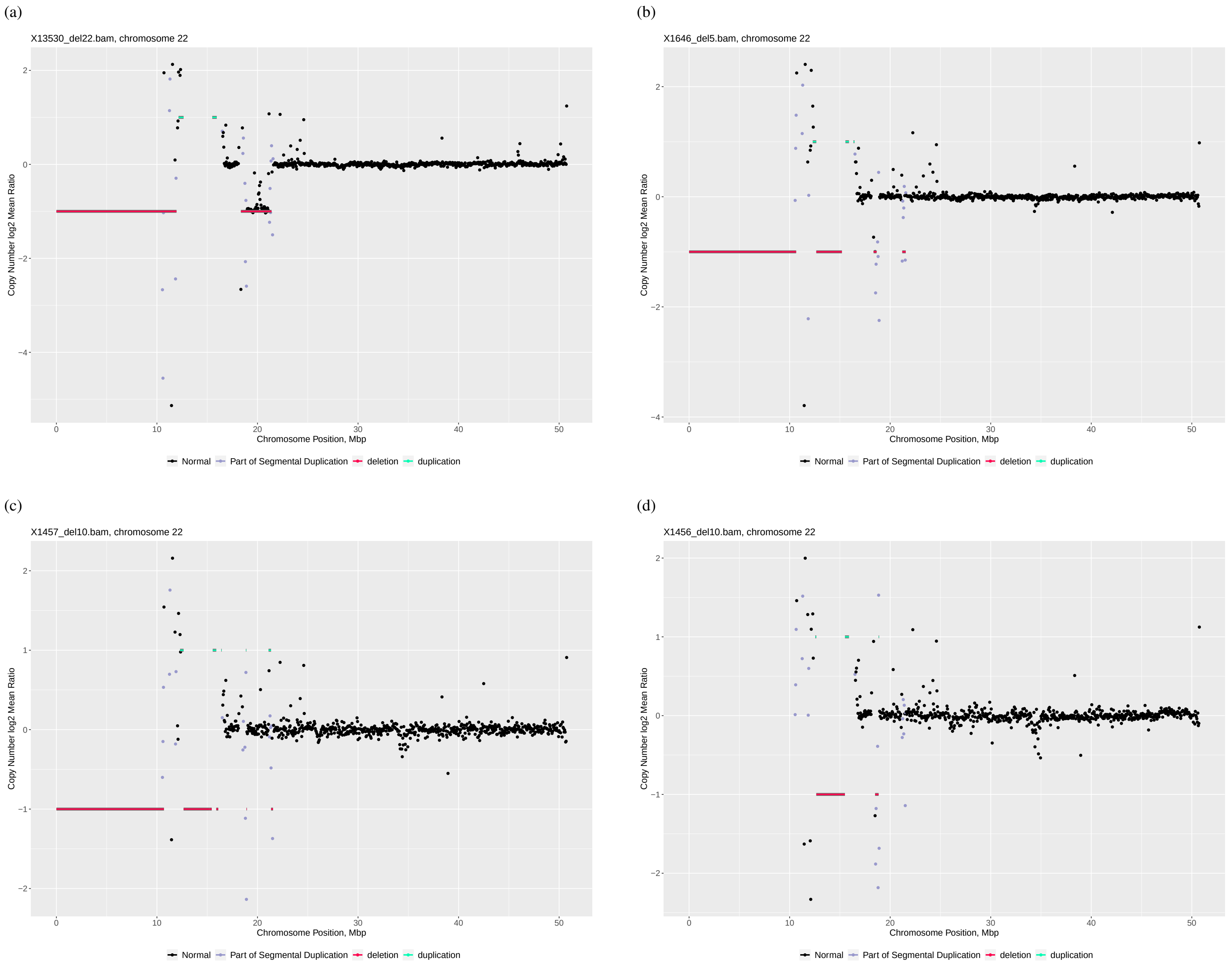

Figure S11: Control-FREEC's output for chromosome 22 in 4 clinical samples. The plot was created using ConanVarvar's native plotting function and precomputed copy number values with real calls from Control-FREEC. All deletions and duplications were assigned the values of –1 and +1 respectively on the logarithmic copy number scale. As shown in (a), Control-FREEC successfully identified the CNV of interest.

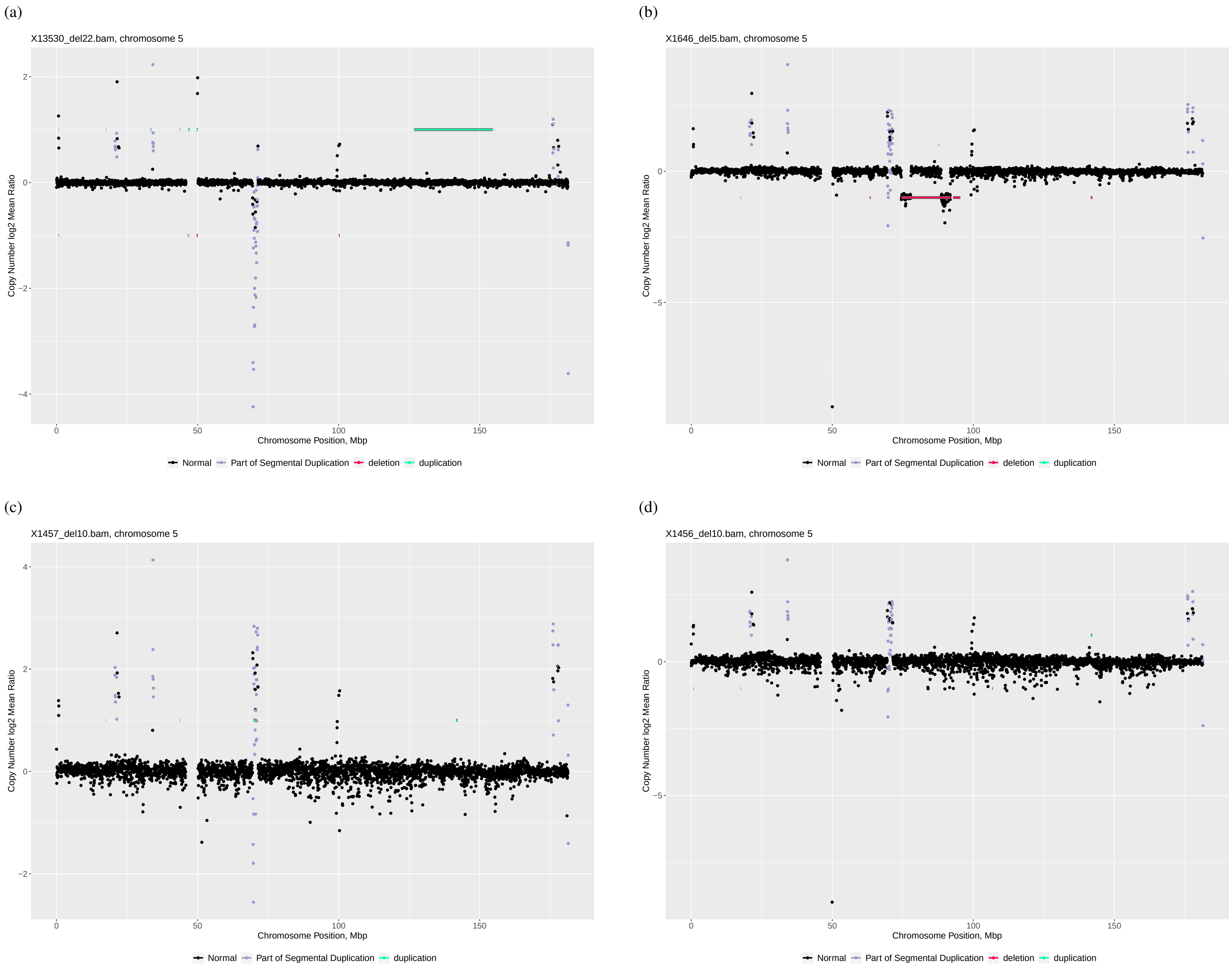

Figure S12: Manta's output for chromosome 5 in 4 clinical samples. The plot was created using ConanVarvar's native plotting function and precomputed copy number values with real calls from Manta. All deletions and duplications were assigned the values of –1 and +1 respectively on the logarithmic copy number scale. As shown in (a) and (b), not only did Manta report the two CNVs of interest as one but it also reported a large false-positive duplication.

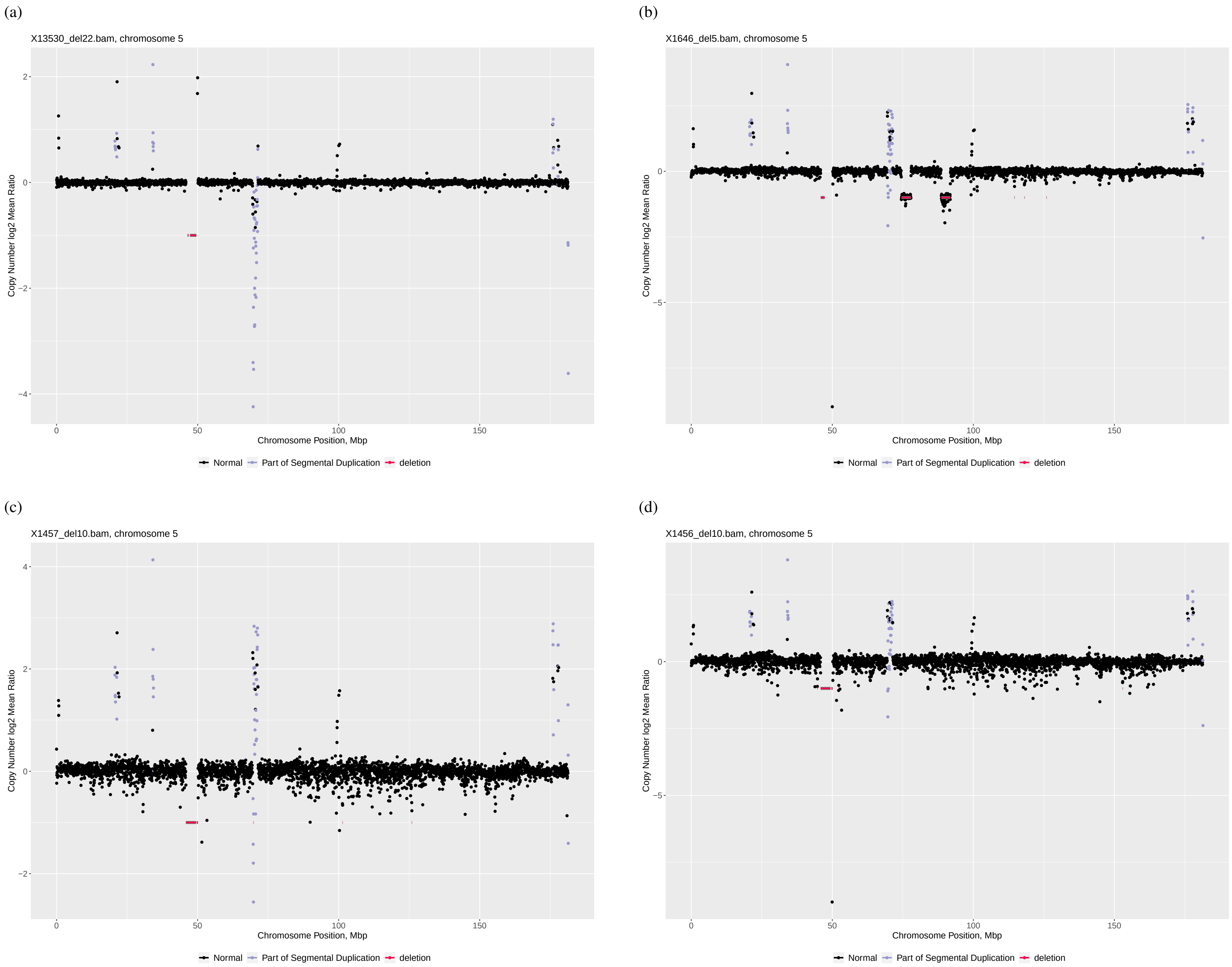

Figure S13: CNVnator's output for chromosome 5 in 4 clinical samples. The plot was created using ConanVarvar's native plotting function and precomputed copy number values with real calls from CNVnator. All deletions and duplications were assigned the values of –1 and +1 respectively on the logarithmic copy number scale. As shown in (b), CNVnator successfully identified the CNVs of interest.

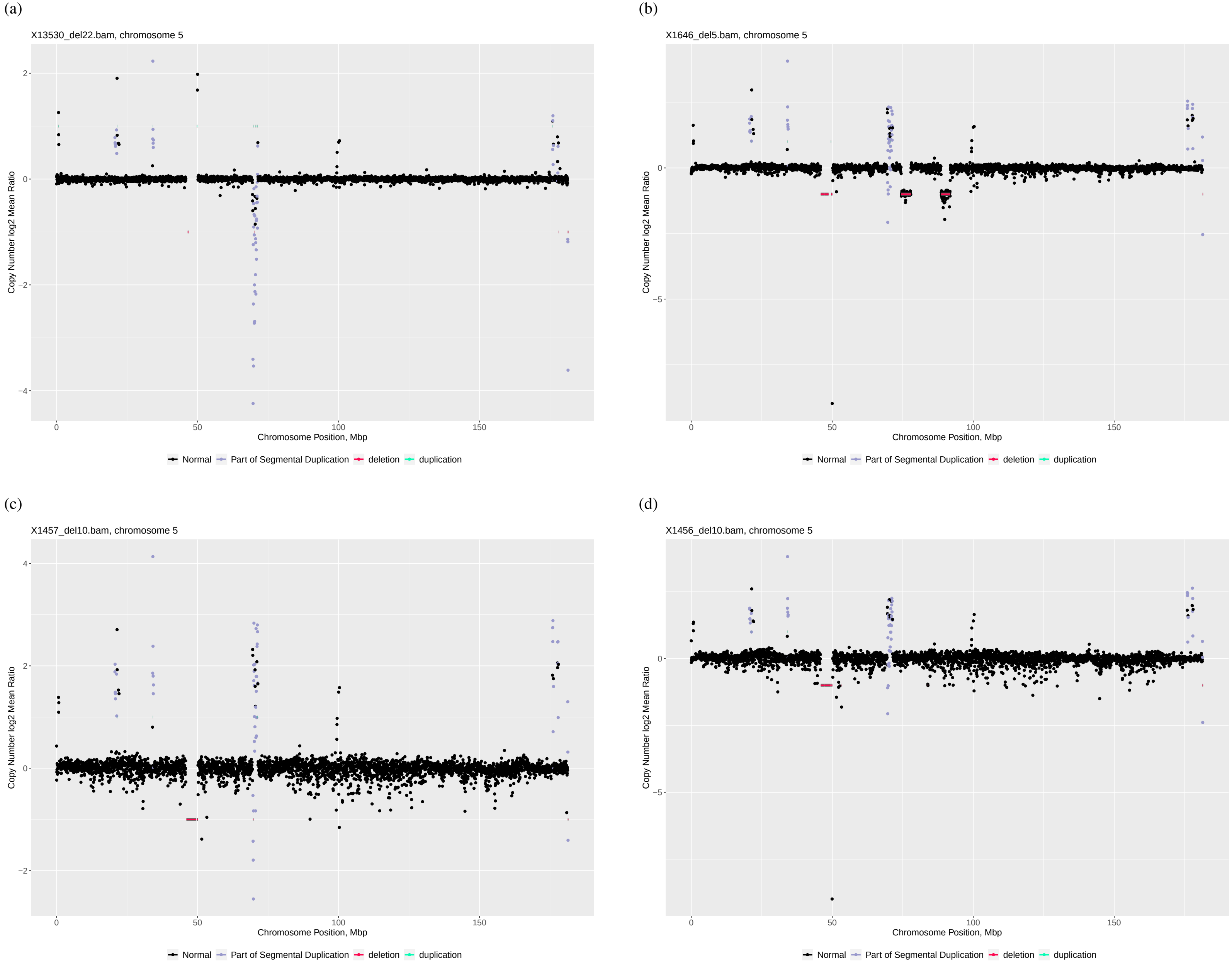

Figure S14: Control-FREEC's output for chromosome 5 in 4 clinical samples. The plot was created using ConanVarvar's native plotting function and precomputed copy number values with real calls from Control-FREEC. All deletions and duplications were assigned the values of –1 and +1 respectively on the logarithmic copy number scale. As shown in (b), Control-FREEC successfully identified the CNVs of interest.

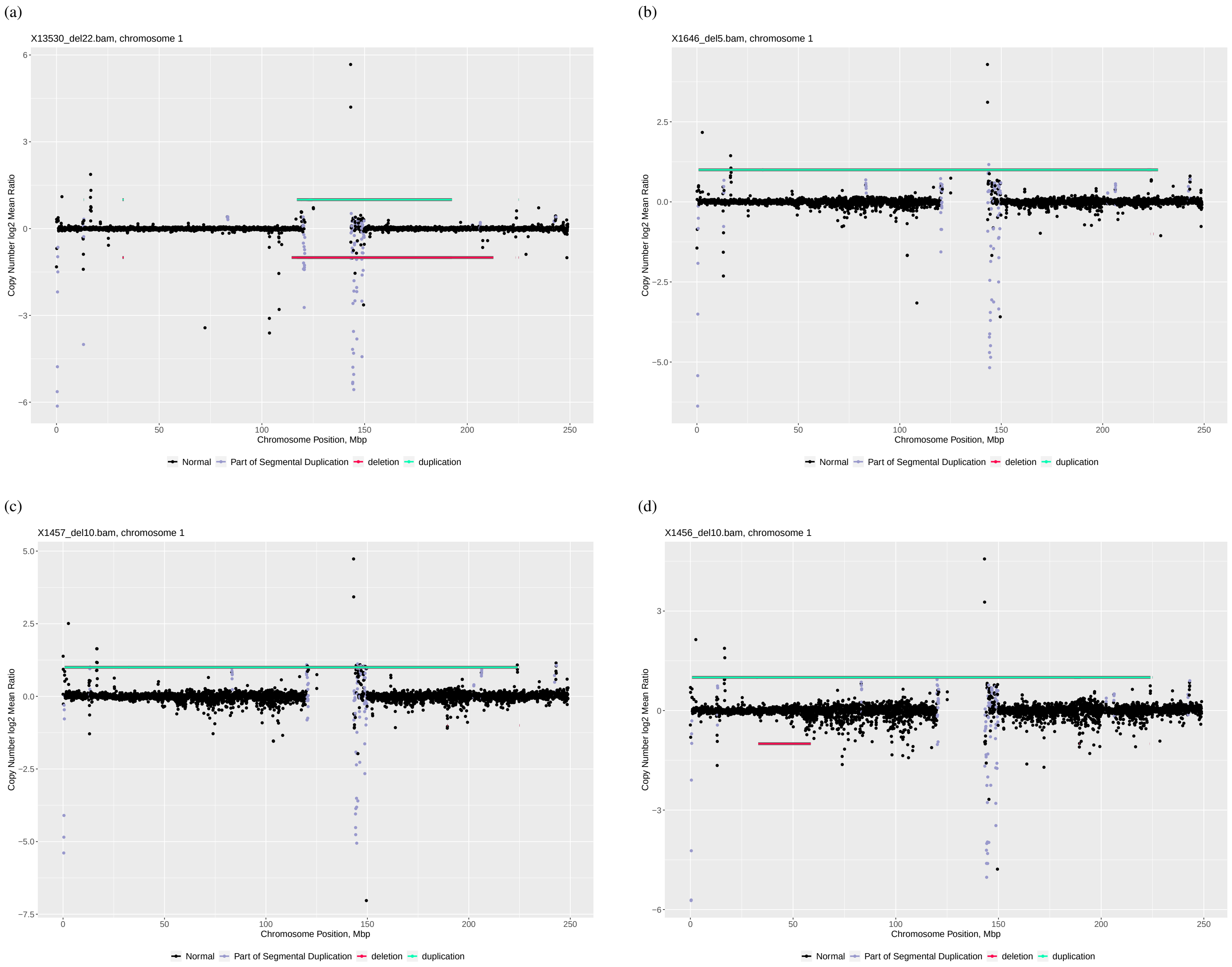

Figure S15: Manta's output for chromosome 1, showing examples of false-positive calls in 4 clinical samples. The plot was created using ConanVarvar's native plotting function and precomputed copy number values with real calls from Manta. All deletions and duplications were assigned the values of –1 and +1 respectively on the logarithmic copy number scale.

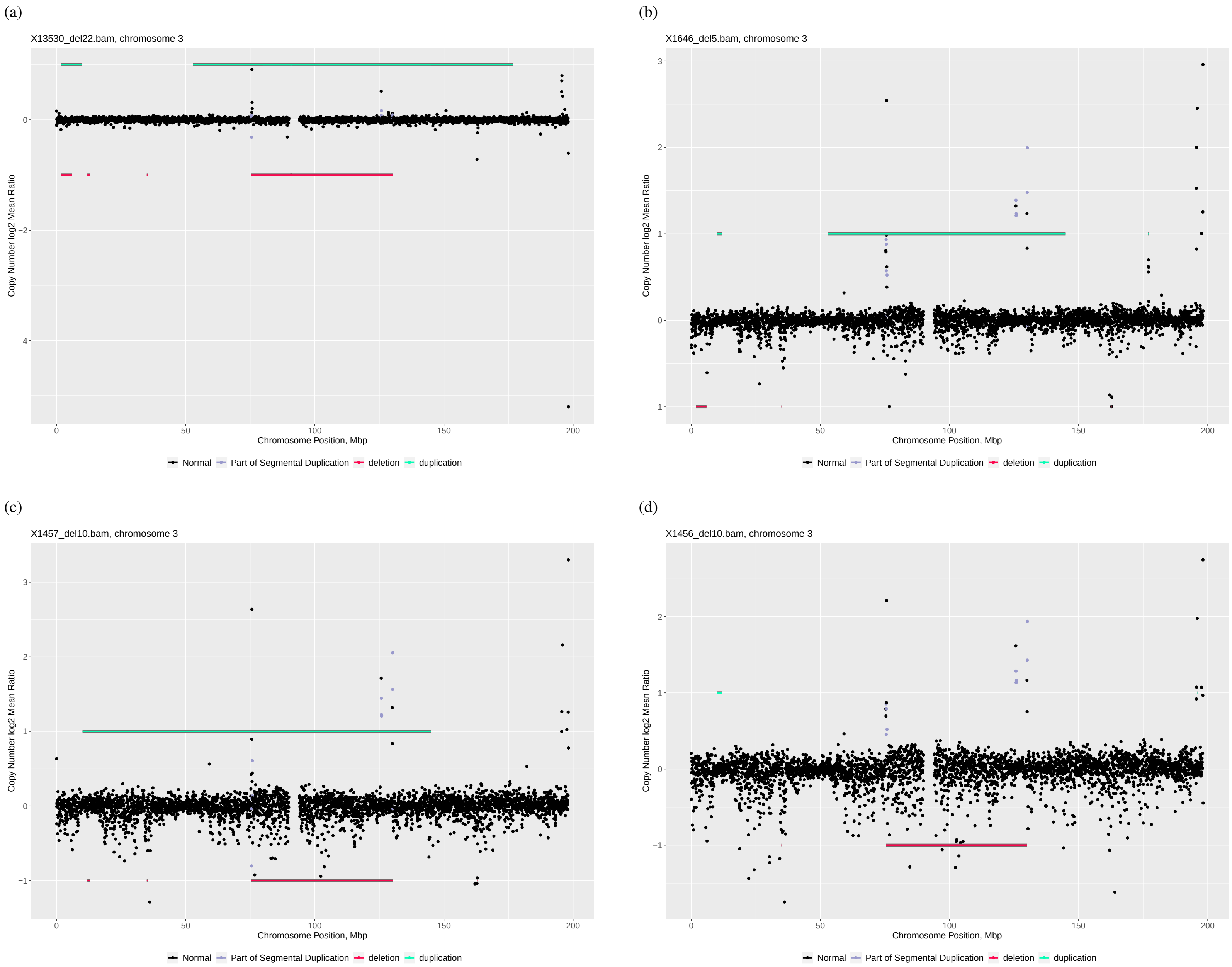

Figure S16: Manta's output for chromosome 3, showing examples of false-positive calls in 4 clinical samples. The plot was created using ConanVarvar's native plotting function and precomputed copy number values with real calls from Manta. All deletions and duplications were assigned the values of –1 and +1 respectively on the logarithmic copy number scale.

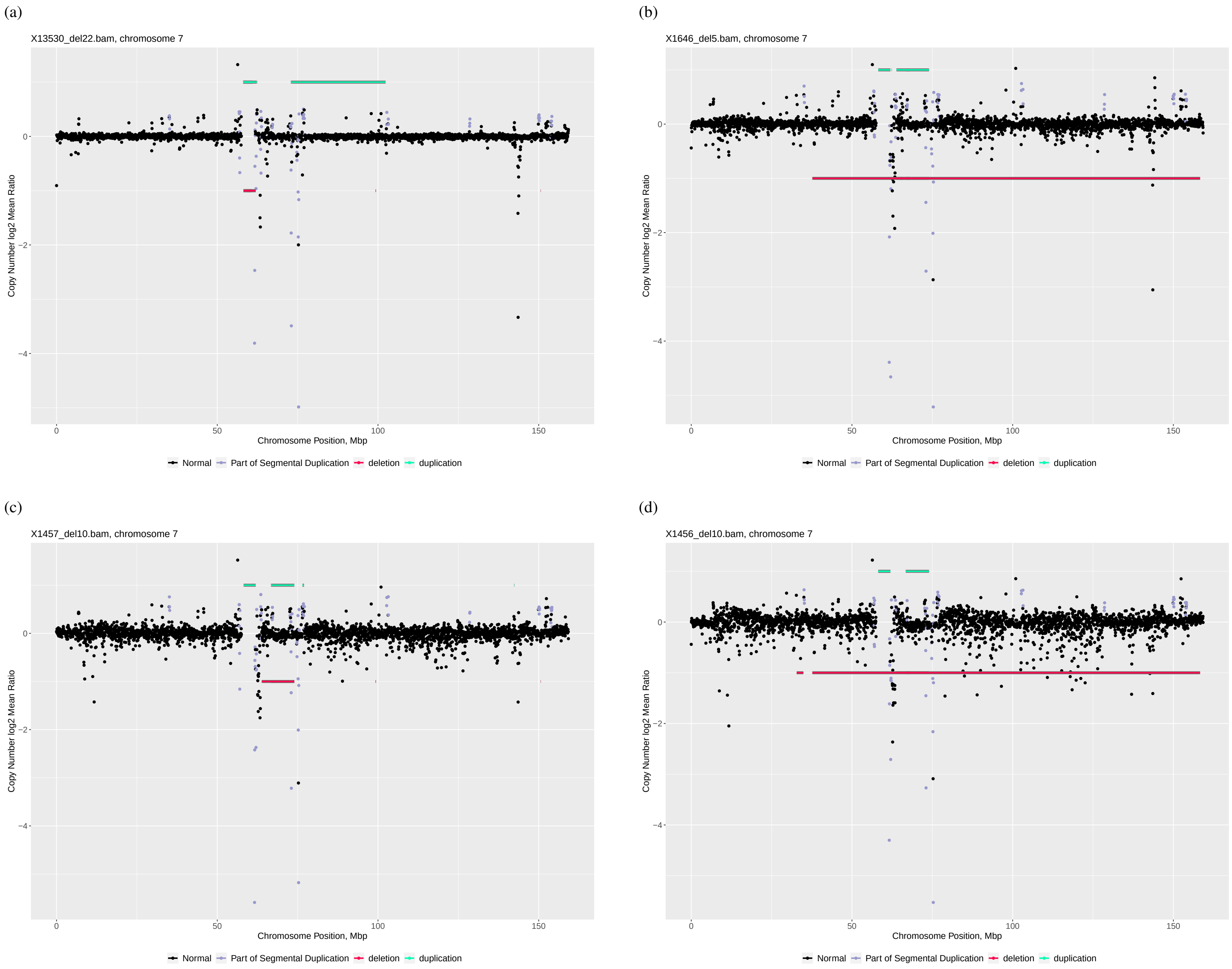

Figure S17: Manta's output for chromosome 7, showing examples of false-positive calls in 4 clinical samples. The plot was created using ConanVarvar's native plotting function and precomputed copy number values with real calls from Manta. All deletions and duplications were assigned the values of –1 and +1 respectively on the logarithmic copy number scale.

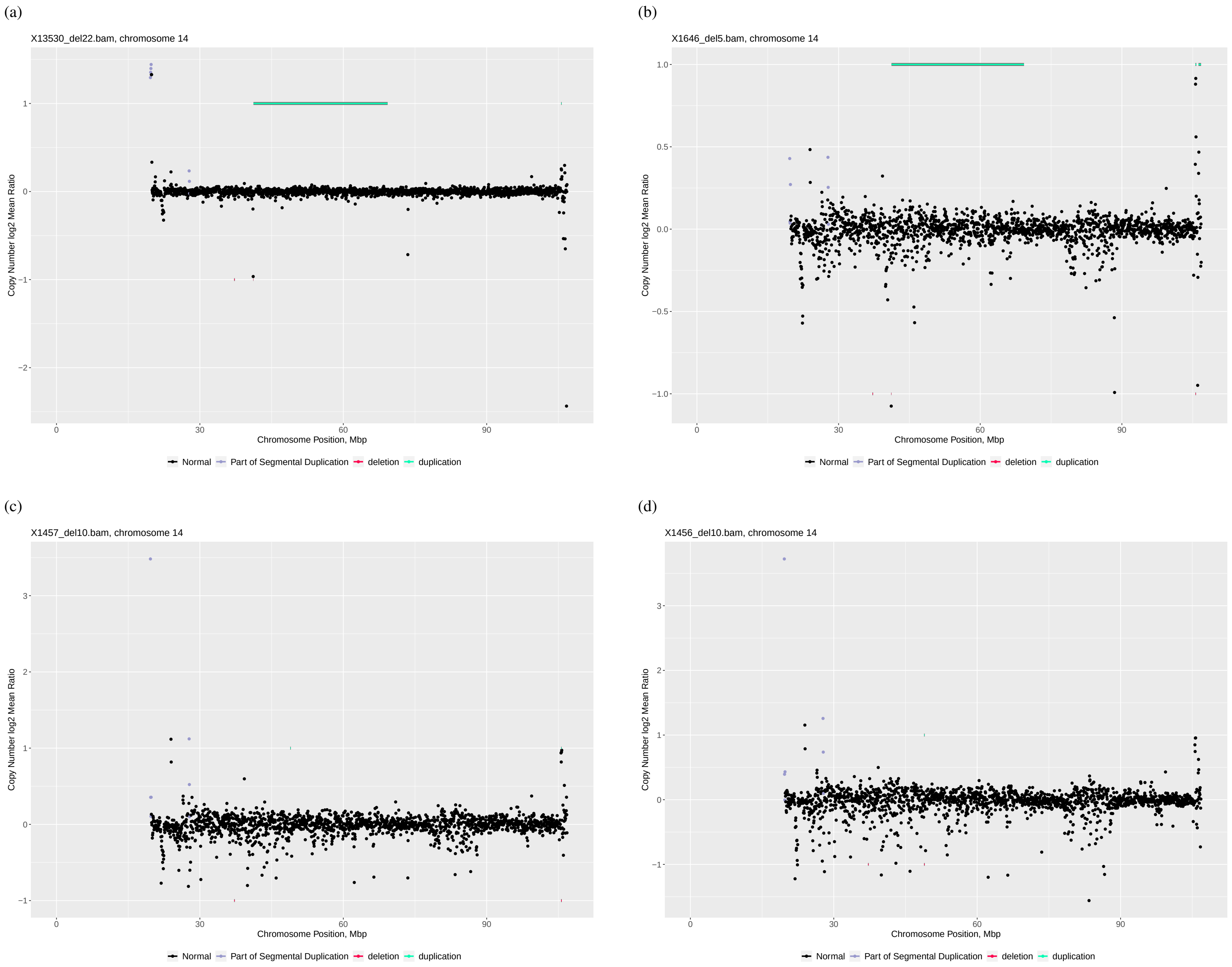

Figure S18: Manta's output for chromosome 14, showing examples of false-positive calls in 4 clinical samples. The plot was created using ConanVarvar's native plotting function and precomputed copy number values with real calls from Manta. All deletions and duplications were assigned the values of –1 and +1 respectively on the logarithmic copy number scale.

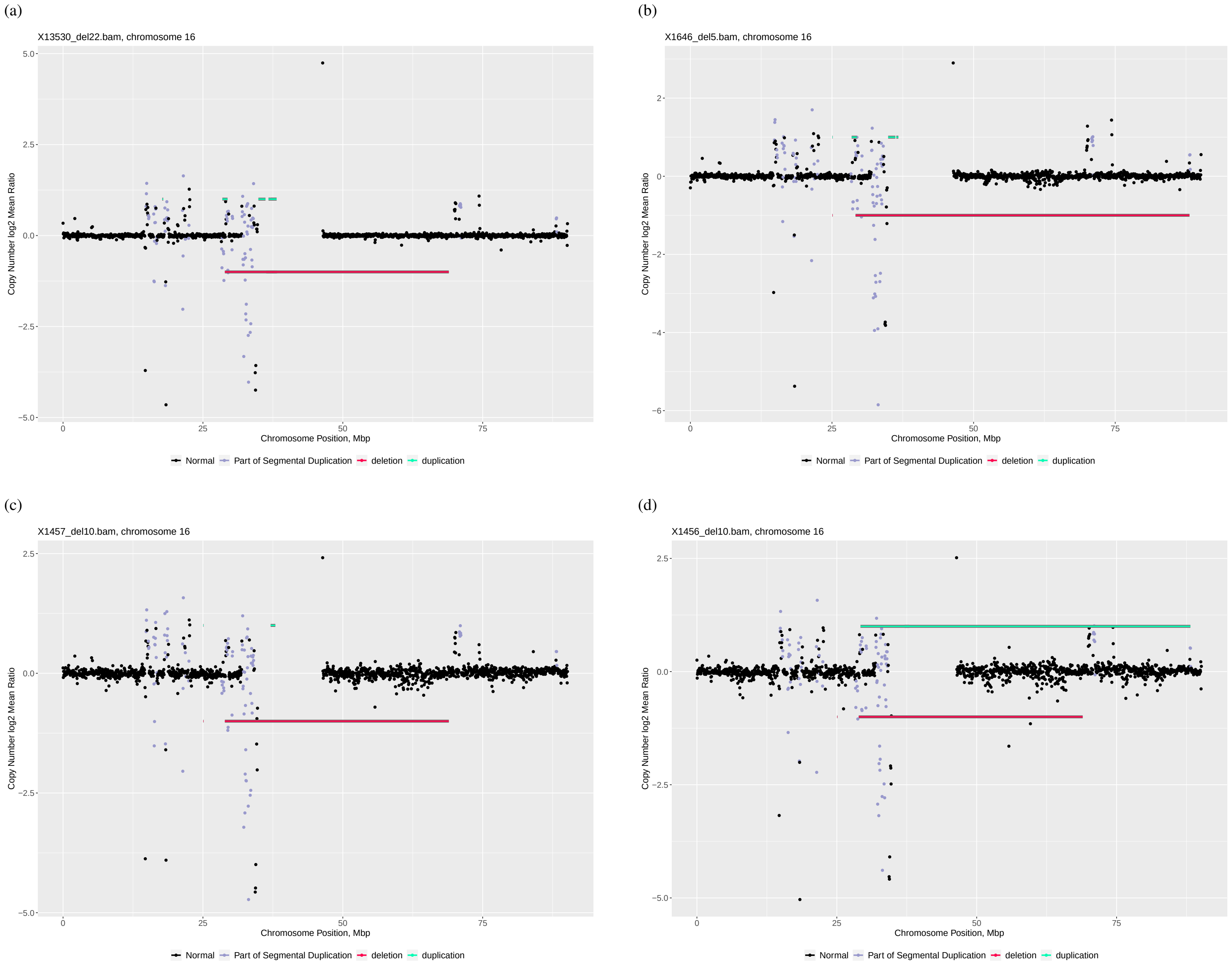

Figure S19: Manta's output for chromosome 16, showing examples of false-positive calls in 4 clinical samples. The plot was created using ConanVarvar's native plotting function and precomputed copy number values with real calls from Manta. All deletions and duplications were assigned the values of –1 and +1 respectively on the logarithmic copy number scale.

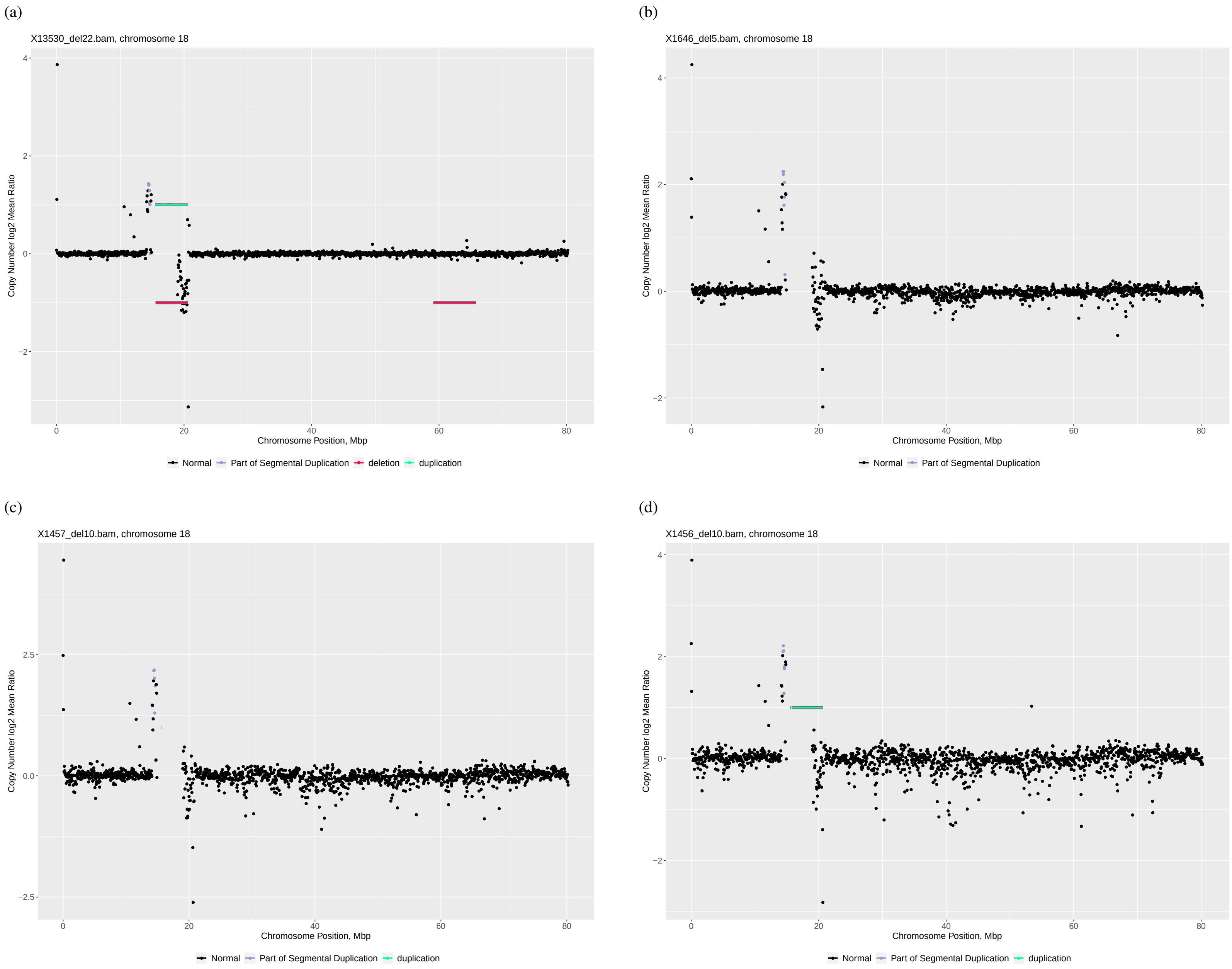

Figure S20: Manta's output for chromosome 18, showing examples of false-positive calls in 4 clinical samples. The plot was created using ConanVarvar's native plotting function and precomputed copy number values with real calls from Manta. All deletions and duplications were assigned the values of –1 and +1 respectively on the logarithmic copy number scale.

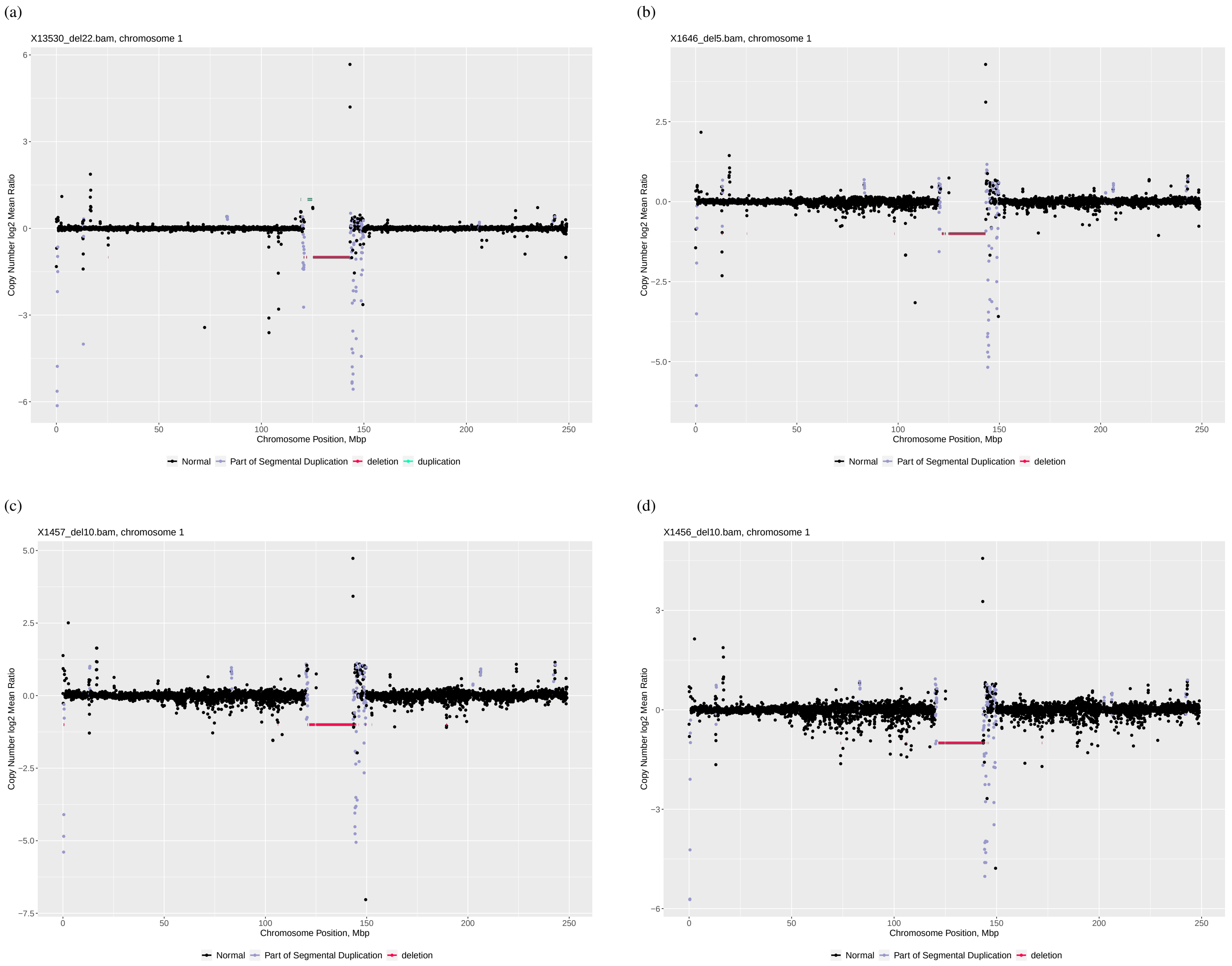

Figure S21: CNVnator's output for chromosome 1, showing examples of false-positive calls in the centromere region in 4 clinical samples. The plot was created using ConanVarvar's native plotting function and precomputed copy number values with real calls from CNVnator. All deletions and duplications were assigned the values of -1 and $+1$ respectively on the logarithmic copy number scale.

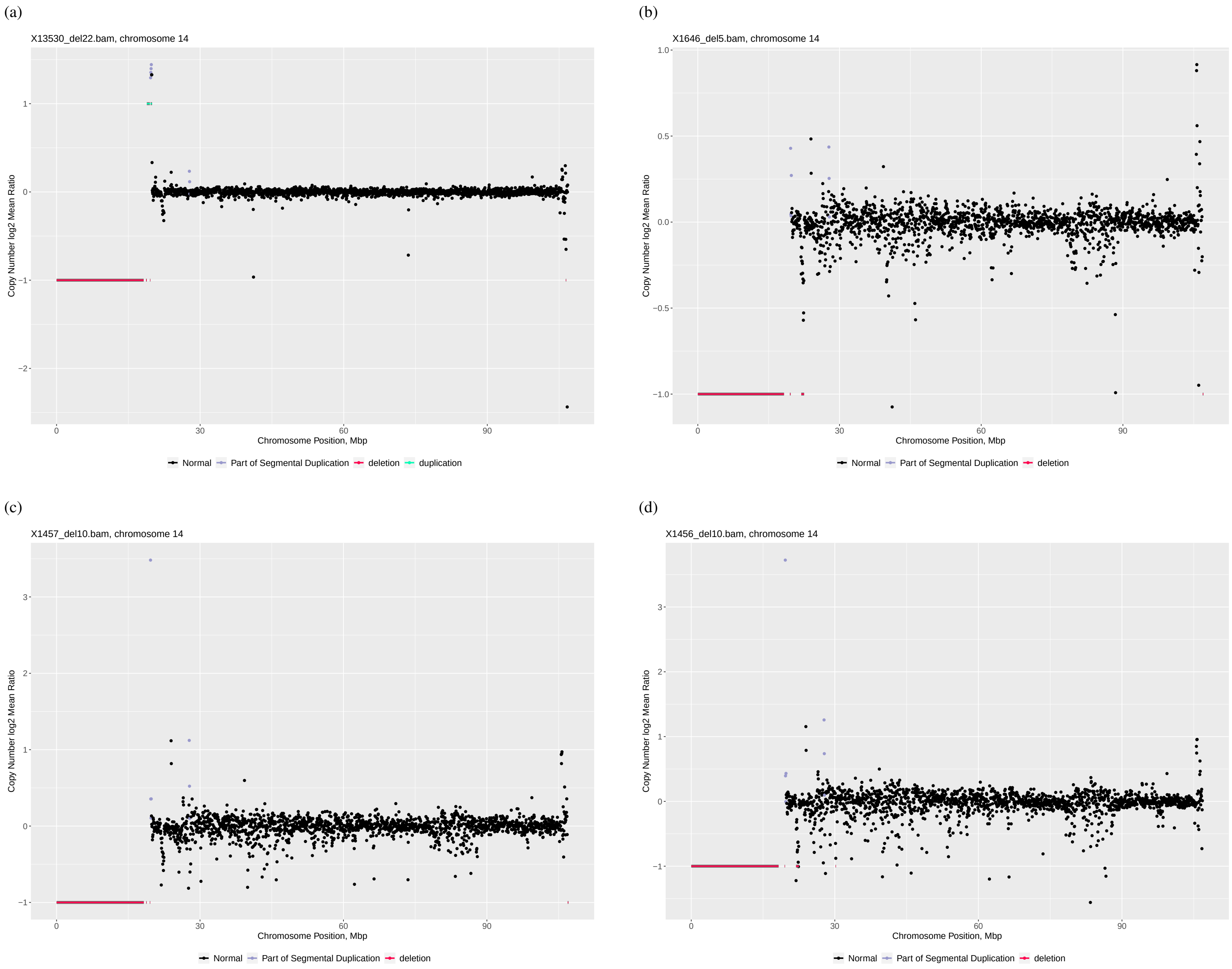

Figure S22: CNVnator's output for chromosome 14, showing examples of false-positive calls in the centromere region in 4 clinical samples. The plot was created using ConanVarvar's native plotting function and precomputed copy number values with real calls from CNVnator. All deletions and duplications were assigned the values of –1 and +1 respectively on the logarithmic copy number scale.

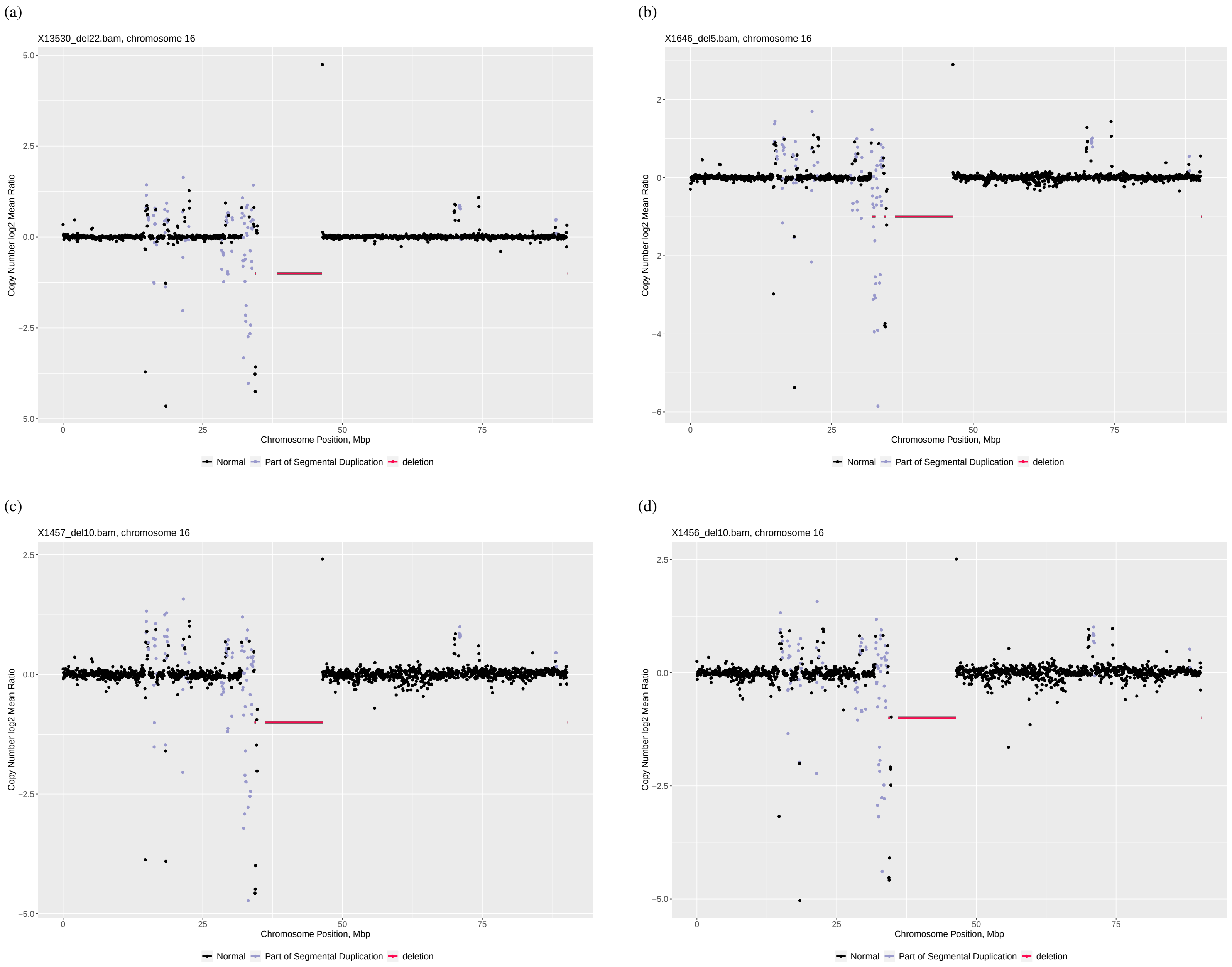

Figure S23: CNVnator's output for chromosome 16, showing examples of false-positive calls in the centromere region in 4 clinical samples. The plot was created using ConanVarvar's native plotting function and precomputed copy number values with real calls from CNVnator. All deletions and duplications were assigned the values of –1 and +1 respectively on the logarithmic copy number scale.

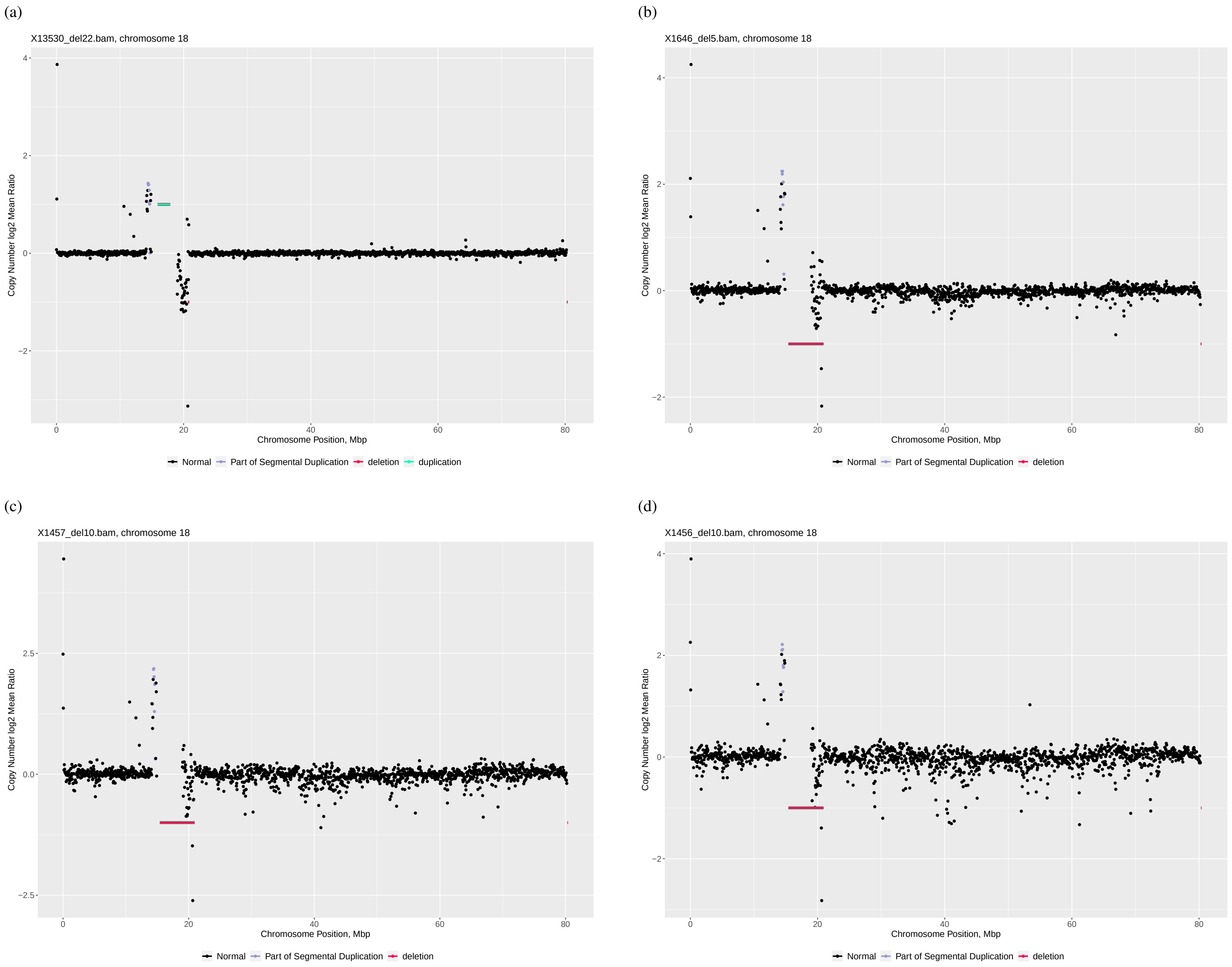

Figure S24: CNVnator's output for chromosome 18, showing examples of false-positive calls in the centromere region in 4 clinical samples. The plot was created using ConanVarvar's native plotting function and precomputed copy number values with real calls from CNVnator. All deletions and duplications were assigned the values of –1 and +1 respectively on the logarithmic copy number scale.

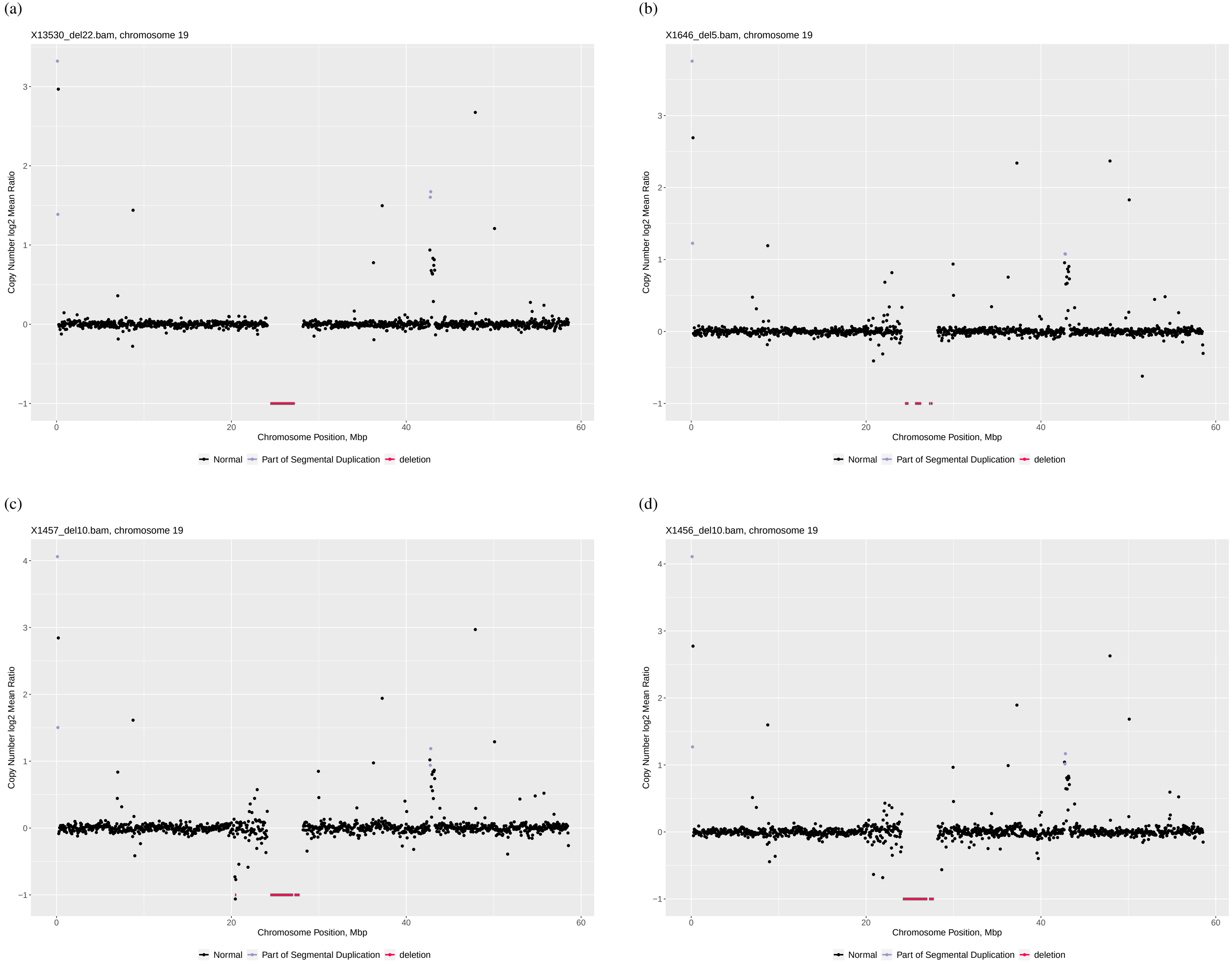

Figure S25: CNVnator's output for chromosome 19, showing examples of false-positive calls in the centromere region in 4 clinical samples. The plot was created using ConanVarvar's native plotting function and precomputed copy number values with real calls from CNVnator. All deletions and duplications were assigned the values of –1 and +1 respectively on the logarithmic copy number scale.

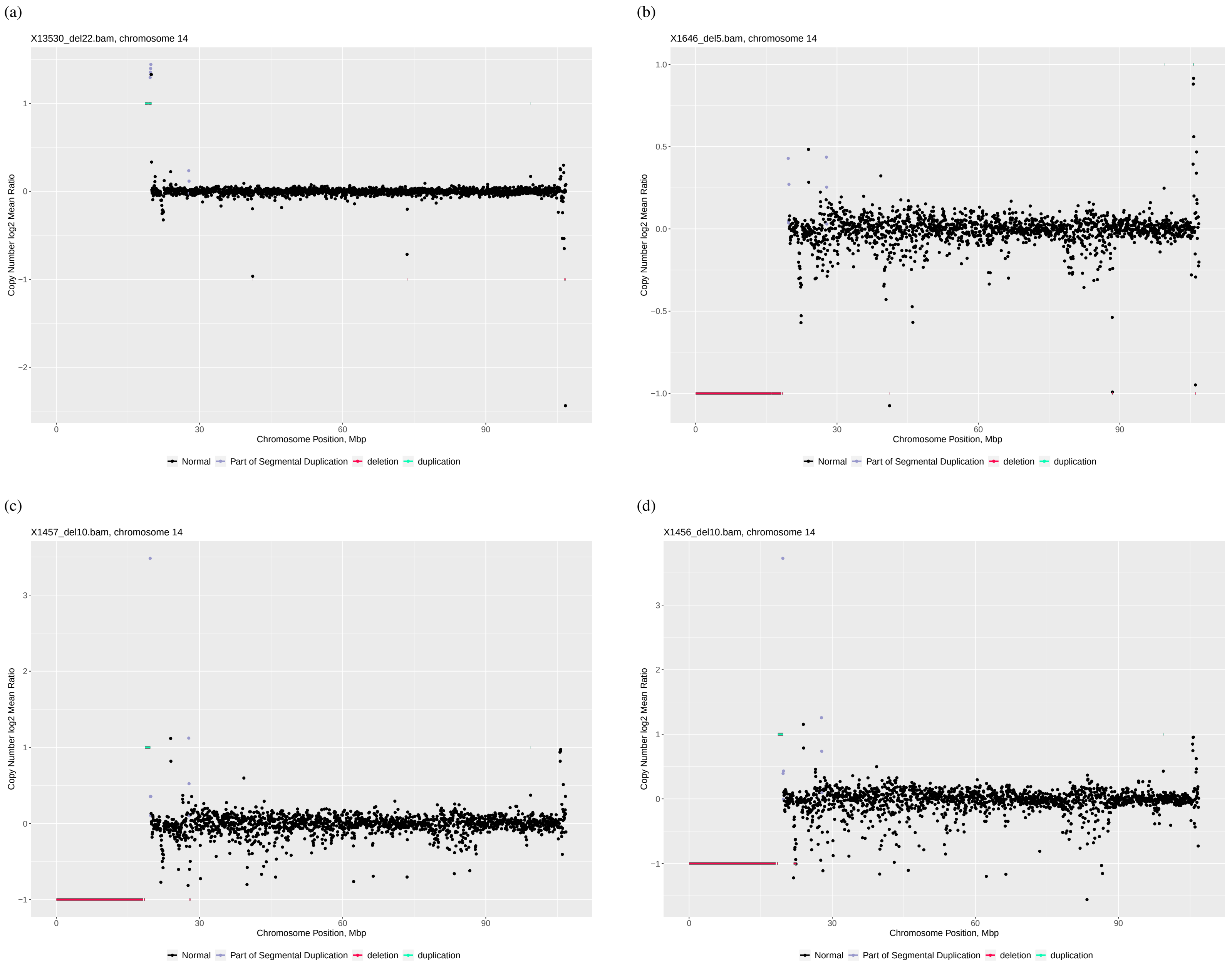

Figure S26: Control-FREEC's output for chromosome 14, showing examples of false-positive calls in the centromere region in 4 clinical samples. The plot was created using ConanVarvar's native plotting function and precomputed copy number values with real calls from Control-FREEC. All deletions and duplications were assigned the values of –1 and +1 respectively on the logarithmic copy number scale.

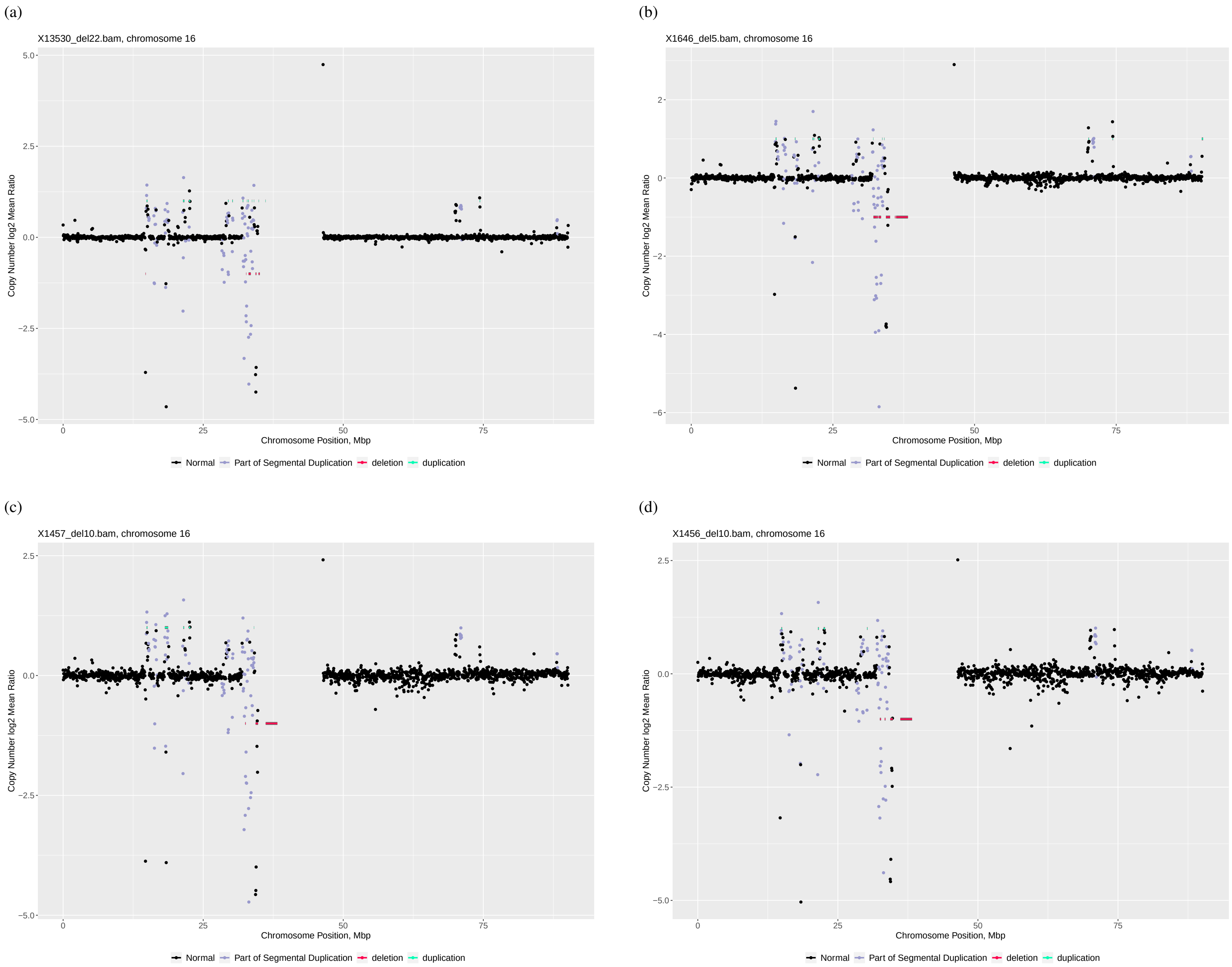

Figure S27: Control-FREEC's output for chromosome 16, showing examples of false-positive calls in the centromere region in 4 clinical samples. The plot was created using ConanVarvar's native plotting function and precomputed copy number values with real calls from Control-FREEC. All deletions and duplications were assigned the values of –1 and +1 respectively on the logarithmic copy number scale.

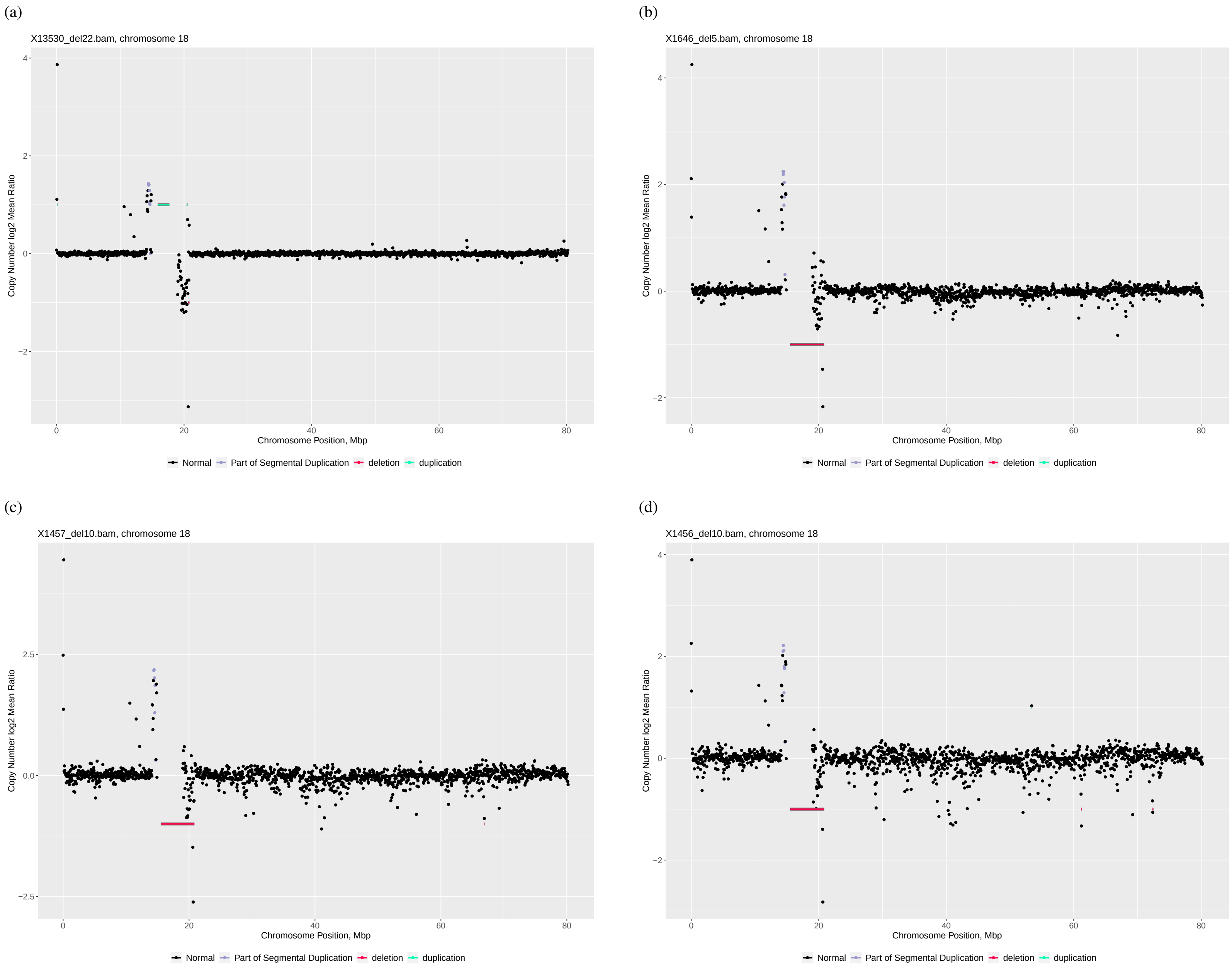

Figure S28: Control-FREEC's output for chromosome 18, showing examples of false-positive calls in the centromere region in 4 clinical samples. The plot was created using ConanVarvar's native plotting function and precomputed copy number values with real calls from Control-FREEC. All deletions and duplications were assigned the values of –1 and +1 respectively on the logarithmic copy number scale.

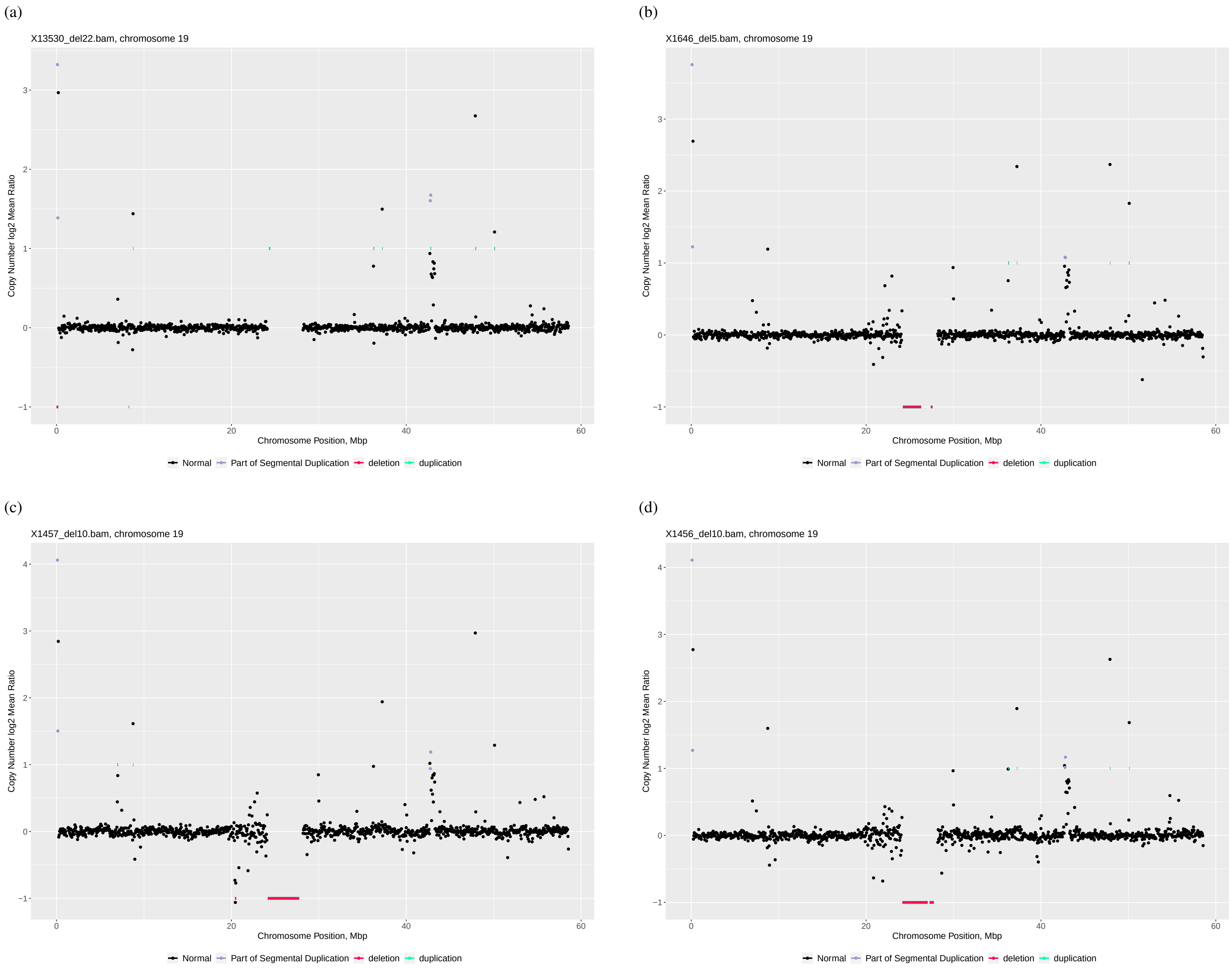

Figure S29: Control-FREEC's output for chromosome 19, showing examples of false-positive calls in the centromere region in 4 clinical samples. The plot was created using ConanVarvar's native plotting function and precomputed copy number values with real calls from Control-FREEC. All deletions and duplications were assigned the values of –1 and +1 respectively on the logarithmic copy number scale.

References

[1] E. Bragin, E. A. Chatzimichali, C. F. Wright, M. E. Hurles, H. V. Firth, A. P. Bevan, and G. J. Swaminathan, "DECIPHER: database for the interpretation of phenotype-linked plausibly pathogenic sequence and copy-number variation," *Nucleic Acids Research*, vol. 42, no. D1, pp. D993–D1000, 2013. DOI: 10.1093/nar/gkt937.

[2] Y. Benjamini and T. P. Speed, "Summarizing and correcting the GC content bias in high-throughput sequencing," *Nucleic Acids Research*, vol. 40, no. 10, e72, 2012. DOI: 10.1093/nar/gks001.

[3] A. Magi, L. Tattini, T. Pippucci, F. Torricelli, and M. Benelli, "Read count approach for DNA copy number variants detection," *Bioinformatics*, vol. 28, no. 4, pp. 470–478, 2012. DOI: 10.1093/bioinformatics/btr707.

[4] S. M. Teo, Y. Pawitan, C. S. Ku, K. S. Chia, and A. Salim, "Statistical challenges associated with detecting copy number variations with next-generation sequencing," *Bioinformatics*, vol. 28, no. 21, pp. 2711–2718, 2012. DOI: 10.1093/bioinformatics/bts535.

[5] G. Klambauer, K. Schwarzbauer, A. Mayr, D.-A. Clevert, A. Mitterecker, U. Bodenhofer, and S. Hochreiter, "Cn.MOPS: mixture of Poissons for discovering copy number variations in next-generation sequencing data with a low false discovery rate," *Nucleic Acids Research*, vol. 40, no. 9, e69, 2012. DOI: 10.1093/nar/gks003.

[6] D. Lai, G. Ha, and S. Sohrab, *HMMcopy: copy number prediction with correction for GC and mappability bias for HTS data*, 2018. [Online]. Available: https://rdrr.io/bioc/HMMcopy/.

[7] A. B. Olshen, E. S. Venkatraman, R. Lucito, and M. Wigler, "Circular binary segmentation for the analysis of array-based DNA copy number data," *Biostatistics*, vol. 5, no. 4, pp. 557–572, 2004. DOI: 10.1093/biostatistics/kxh008.

[8] P. Baldi and A. D. Long, "A Bayesian framework for the analysis of microarray expression data: regularized t-test and statistical inferences of gene changes," *Bioinformatics*, vol. 17, no. 6, pp. 509–519, 2001. DOI: 10.1093/bioinformatics/17.6.509.

[9] A. A. Fodor, T. L. Tickle, and C. Richardson, "Towards the uniform distribution of null P values on affymetrix microarrays," *Genome Biology*, vol. 8, no. 5, R69, 2007. DOI: 10.1186/gb-2007-8-5-r69.

[10] A. Y. Lee, A. D. Ewing, K. Ellrott, Y. Hu, K. E. Houlahan, J. C. Bare, S. M. G. Espiritu, V. Huang, K. Dang, Z. Chong, C. Caloian, T. N. Yamaguchi, M. R. Kellen, K. Chen, T. C. Norman, S. H. Friend, J. Guinney, G. Stolovitzky, D. Haussler, A. A. Margolin, J. M. Stuart, and P. C. Boutros, "Combining accurate tumor genome simulation with crowdsourcing to benchmark somatic structural variant detection," *Genome Biology*, vol. 19, no. 1, p. 188, 2018. DOI: 10.1186/s13059-018-1539-5.

[11] T. Kuo, M. C. Frith, J. Sese, and P. Horton, "EAGLE: Explicit Alternative Genome Likelihood Evaluator," *BMC Medical Genomics*, vol. 11, no. 28, 2018. DOI: 10.1186/s12920-018-0342-1.
